# Data processing of qualitative results from an interlaboratory comparison for the detection of “Flavescence dorée” phytoplasma: How the use of statistics can improve the reliability of the method validation process in plant pathology

**DOI:** 10.1371/journal.pone.0175247

**Published:** 2017-04-06

**Authors:** Aude Chabirand, Marianne Loiseau, Isabelle Renaudin, Françoise Poliakoff

**Affiliations:** 1 Unit for Tropical Pests and Diseases, Plant Health Laboratory (LSV), French Agency for Food, Environmental and Occupational Health & Safety (ANSES), Saint-Pierre, Reunion Island, France; 2 Plant Health Laboratory (LSV), French Agency for Food, Environmental and Occupational Health & Safety (ANSES), Angers, France; Academia Sinica, TAIWAN

## Abstract

A working group established in the framework of the EUPHRESCO European collaborative project aimed to compare and validate diagnostic protocols for the detection of “Flavescence dorée” (FD) phytoplasma in grapevines. Seven molecular protocols were compared in an interlaboratory test performance study where each laboratory had to analyze the same panel of samples consisting of DNA extracts prepared by the organizing laboratory. The tested molecular methods consisted of universal and group-specific real-time and end-point nested PCR tests. Different statistical approaches were applied to this collaborative study. Firstly, there was the standard statistical approach consisting in analyzing samples which are known to be positive and samples which are known to be negative and reporting the proportion of false-positive and false-negative results to respectively calculate diagnostic specificity and sensitivity. This approach was supplemented by the calculation of repeatability and reproducibility for qualitative methods based on the notions of accordance and concordance. Other new approaches were also implemented, based, on the one hand, on the probability of detection model, and, on the other hand, on Bayes’ theorem. These various statistical approaches are complementary and give consistent results. Their combination, and in particular, the introduction of new statistical approaches give overall information on the performance and limitations of the different methods, and are particularly useful for selecting the most appropriate detection scheme with regards to the prevalence of the pathogen. Three real-time PCR protocols (methods M4, M5 and M6 respectively developed by Hren (2007), Pelletier (2009) and under patent oligonucleotides) achieved the highest levels of performance for FD phytoplasma detection. This paper also addresses the issue of indeterminate results and the identification of outlier results. The statistical tools presented in this paper and their combination can be applied to many other studies concerning plant pathogens and other disciplines that use qualitative detection methods.

## Introduction

The use of analytical methods capable of producing reliable analytical results is a prerequisite to the effective control of quarantine plant pathogens. Consequently, it is relevant to evaluate and test the methods to define how valid and reliable the produced results are for an intended purpose, *i*.*e*. to validate the methods.

Validation is defined in the ISO/IEC 17025 standard [1] as “the confirmation by examination and the provision of objective evidence that the particular requirements for a specific intended use are fulfilled”. A rigorous validation process includes both single laboratory validation and an interlaboratory test performance study (TPS), the latter also being referred to as a ring test or collaborative trial [2].

An interlaboratory test performance study is considered to be a more reliable indicator of test performance when used in other laboratories because it requires testing of the method in multiple laboratories, by different analysts using different reagents, supplies and equipment and working in different laboratory environments. Interlaboratory test performance studies are an essential part of the validation process for analytical methods. The purposes of an interlaboratory test performance study are to determine the performance of one or more tests among laboratories, to estimate the reproducibility of the test(s), and if several tests included, to establish their comparability. In this context the use of statistics, both for the design of interlaboratory test performance studies and the processing of the participants’ results, is essential to make sure that differences observed between tests have a high probability of being real and not due to random effect.

As a French National Reference Laboratory for plant pathology, the ANSES Plant Health Laboratory organizes interlaboratory test performance studies in order to ensure that the methods used by officially approved French laboratories (certified by government services) are capable of producing reliable analytical results for the detection of plant pathogens. In this context, the ANSES Plant Health Laboratory participated in a working group established in the framework of EUPHRESCO (EUropean PHytosanitary RESearch Coordination, a phytosanitary European Research Area Network (ERA-NET) project) which aimed to compare and validate diagnostic protocols for the detection of “Flavescence dorée” (FD) phytoplasma in grapevines. FD is one of the main grapevine diseases in Europe included in the European legislation as a quarantine pest (Directive 2000/29/EC). Seven methods based on conventional and real-time PCR for the detection of this phytoplasma were selected and subject to interlaboratory trials performed in 14 European laboratories.

Interlaboratory test performance studies in plant pathology have a number of notable features including the processing of qualitative results. This paper focuses on different statistical tools that can be used to process the results of an interlaboratory test performance study and presents their application to the collaborative study on the detection of FD phytoplasma. The use of statistics in data processing helps to distinguish between differences that are due to chance and those that probably indicate a real effect of the evaluated methods and consequently ensures the reliability of results and the related decision-making process. Standard statistical approaches based on the PM7/76(2) [3], PM7/98(2) [4] and PM7/122(1) [2] standards and on the ISO 16140 standard [5] first made it possible to determine traditional performance criteria such as analytical specificity, analytical sensitivity, repeatability, reproducibility, concordance odds ratio and their associated confidence intervals. Different statistical tests were then implemented to identify differences in these performance criteria between methods. This standard approach was supplemented by two new statistical approaches in plant pathology: the probability of detection model, which is, to our knowledge, published for the first time for plant pathology diagnostic protocols and the Bayesian approach in line with recent publications in plant pathology [6–9].

## Materials and methods

### Study design

The interlaboratory test performance study was conducted in two stages: (i) the evaluation of the analytical specificity of the methods, and (ii) the evaluation of their analytical sensitivity, repeatability and reproducibility, and application of the probability of detection model.

The TPS involved 14 partner laboratories in ten European countries for the first stage and a sub-group of five partner laboratories in five European countries for the second stage. Detailed information on the participating laboratories is available in S1 Table.

During the TPS, for the evaluation of the different methods, the participating laboratories had to analyze identical series of blinded samples according to the working protocols and data reporting sheets provided and using the same plate plans. The DNA extracts were to be amplified in two tubes in accordance with the standards for molecular biology methods.

#### Tested methods

Seven molecular methods were subject to interlaboratory trials: 1) method Ma: a universal nested-PCR assay followed by RFLP analysis with TaqI [10]; 2) methods M1 and M2: two group-specific nested-PCR assays [11–14]; 3) methods M3 and M4: two real-time PCR assays for specific detection of 16SrV group phytoplasmas [15, 16]; 4) methods M5 and M6: two real-time PCR assays for co-detection of 16SrV and 16SrXII phytoplasma groups (groups containing FD and Bois noir (BN) phytoplasmas respectively) and also including an internal grapevine control ([17] and oligonucleotides under-patent IPADLAB). Table 1 presents the methods evaluated during this ring-test. Details are given for each method (amplification conditions) in S2 and S3 Tables.

**Table 1:** Methods evaluated during the interlaboratory test performance study.

Methods			M1	M2	Ma	M3	M4	M5	M6
**Type of amplification**			End-point PCR	End-point PCR	End-point PCR	Real-time PCR	Real-time PCR	Real-time PCR	Real-time PCR
			Nested-PCR	Duplex nested-PCR	Nested-PCR followed by RFLP analysis	Simplex PCR	Simplex PCR	Triplex PCR	Triplex PCR
**Targeted area of the genome**			16S rDNA	SecY gene	16S rDNA	16S rDNA	SecY gene	map gene	unknown
**Bibliographical references**			[11, 14]	[18] [13, 15]	[11]	[15]	[16]	[17]	Under-patent (IPADLAB)
**Evaluation of analytical specificity**	**Number of laboratories**^**a**^		14	12 (13)^c^	5 (6)^c^	7	10	7 (8)^c^	9
	**Number of results**^**a**^^**b**^	**overall**	336	288 (312)	120 (144)	168	240	168 (192)	216
		**from positive samples**	210	180 (195)	75 (90)	105	150	105 (120)	135
		**from negative samples**	126	108 (117)	45 (54)	63	90	63 (72)	81
	**Number of indeterminate results**^**a**^	**overall**	16	11 (11)	3 (3)	4	8	5 (11)	14
		**from positive samples**	9	8 (8)	2 (2)	0	3	2 (4)	5
		**from negative samples**	7	3 (3)	1 (1)	4	5	3 (7)	9
	**Rate of indeterminate results (%)**^**a**^	**overall**	4.76	3.82 (3.53)	2.50 (2.08)	2.38	3.33	2.98 (5.73)	6.48
		**from positive samples**	4.29	4.44 (4.10)	2.67 (2.22)	0.00	2.00	1.90 (3.33)	3.70
		**from negative samples**	5.56	2.78 (2.56)	2.22 (1.85)	6.35	5.56	4.76 (9.72)	10.23
**Evaluation of analytical sensitivity, repeatability and reproducibility**	**Number of laboratories**		5	5	2	5	5	5	5
	**Number of results**		375	375	150	375	375	375	375

^a^The number in brackets indicates the value without exclusion of data

^b^The number of results was calculated as follows: number of laboratories x number of results per method and laboratory (overall, from positive samples and from negative samples). The number of results per method and laboratory from positive samples was 15; the number of results per method and laboratory from negative samples was 9 and the number of overall results per method and laboratory was 24

^c^Exclusion of results of P6 for method M2, results of P5 for method Ma and results of P9 for method M5 (considered as outliers, for more details: see “outlier results” section)

Not all the methods were implemented by all the participants. S4 Table summarizes which methods were implemented by which participant at each stage of the TPS.

Prior to the implementation of the TPS, partners who were involved in the evaluation of the real-time PCR methods were invited to determine the cut-off value. To do so, they received the same batch of samples which consisted in a serial dilution (ten levels) of one positive DNA extract in healthy grapevine DNA and one DNA extract from healthy grapevines.

It is worth noting that several laboratories had planned to implement method Ma but could not provide results with this method (no amplification was obtained from the positive controls), whereas results were provided for all the other methods these laboratories had planned to implement.

#### Samples

Positive and negative reference materials obtained from different reference collections were used to prepare the TPS samples. The samples sent to the TPS participants consisted of DNA extracts. To evaluate analytical specificity (first stage of the evaluation), 24 blind samples (Table 2) were analyzed by each participant: these samples consisted of 15 samples positive for phytoplasmas of the 16SrV group (obtained from different parts of Europe in order to have a wide diversity of strains) and nine samples negative for phytoplasmas of the 16SrV group. Almost no methods are able to distinguish between the FD phytoplasma sub-groups; therefore, the performance of methods was calculated regarding their ability to detect all 16SrV phytoplasmas and distinguish them from phytoplasmas in other ribosomal groups. In detail, the 15 positive samples included 11 grapevine samples positive for FD phytoplasma and four samples positive for other phytoplasmas of the 16SrV group. The nine negative samples included four samples of healthy *Vitis vinifera* and five samples contaminated by phytoplasmas from groups other than the 16SrV group.

**Table 2:** Samples used to evaluate analytical specificity.

Sample codes	Origins	Details	16SrV status^a^	Mean Ct values^b^
**a**	JKI Germany	Palatinate grapevine yellows 16SrV-C	1	19.04
**b**	DipSA USA	Aster yellows 16SrI-B	0	-
**c**	ANSES France	‘*Candidatus* Phytoplasma solani’ 16SrXII-A	0	-
**d**	CRA-PAV Italy	Healthy grapevine	0	-
**e**	DipSA USA	‘*Candidatus* Phytoplasma fraxini’ 16SrVII	0	-
**f**	DipSA Italy	Flavescence dorée phytoplasma FD-C	1	27.97
**g**	AGES Austria	Flavescence dorée phytoplasma FD-C	1	24.69
**h**	ANSES France	‘*Candidatus* Phytoplasma solani’ 16SrXII-A	0	-
**i**	INRB Portugal	Flavescence dorée phytoplasma FD-D	1	24.49
**j**	DipSA Italy	*Rubus* Stunt phytoplasma 16SrV-E	1	24.69
**k**	ANSES France	Mix of healthy grapevine	0	-
**l**	NIB Slovenia	Flavescence dorée phytoplasma FD-D	1	26.68
**m**	ANSES France	Flavescence dorée phytoplasma	1	25.05
**n**	ANSES France	Flavescence dorée phytoplasma and ‘*Candidatus* Phytoplasma solani’	1	26.81
**o**	ANSES France	Flavescence dorée phytoplasma	1	28.35
**p**	ACW Switzerland	Flavescence dorée phytoplasma	1	23.58
**q**	NIB Slovenia	Healthy grapevine	0	-
**r**	DipSA Italy	Grapevine yellows phytoplasma 16SrIII-B	0	-
**s**	DipSA China	Jujube witches’ broom phytoplasma 16SrV-B	1	31.05
**t**	ANSES France	Flavescence dorée phytoplasma	1	26.10
**u**	DipSA Europe	Elm witches’ broom phytoplasma 16SrV-A	1	**-**
**v**	ANSES France	Mix of healthy grapevine	0	-
**w**	IPEP Serbia	Flavescence dorée phytoplasma	1	19.76
**x**	ANSES France	Flavescence dorée phytoplasma	1	31.95

^a^ The 16SrV phytoplasma group is the target of each detection method because none of the evaluated methods is able to distinguish between FD phytoplasma and the other phytoplasmas of the same group

^b^ Mean Ct value: 6 assays (except for sample “x”: 5 assays and sample “i": 4 assays).

To evaluate analytical sensitivity, repeatability and reproducibility, and to apply the probability of detection model (second stage of the evaluation), five-fold serial dilutions for three FD-positive samples (Table 3) were analyzed five times each.

**Table 3:** Samples used to evaluate analytical sensitivity, repeatability and reproducibility.

Sample codes	Samples used for the dilutions^a^	Dilution levels	16SrV status^b^
**A1**	Sample p	1.0·10^−1^	1
**A2**		1.0·10^−2^	1
**A3**		3.0·10^−3^	1
**A4**		1.1·10^−3^	1
**A5**		3.7·10^−4^	1
**B1**	Sample x	1.0·10^−1^	1
**B2**		1.0·10^−2^	1
**B3**		3.0·10^−3^	1
**B4**		1.1·10^−3^	1
**B5**		3.7·10^−4^	1
**C1**	Sample w	1.0·10^−1^	1
**C2**		1.0·10^−2^	1
**C3**		3.0·10^−3^	1
**C4**		1.1·10^−3^	1
**C5**		3.7·10^−4^	1

^a^ In reference to samples listed in Table 2.

^b^ The 16SrV phytoplasma group is the target of each detection method because none of the evaluated methods is able to distinguish between FD phytoplasma and other phytoplasmas of the same group

Details on the samples used at each step of the TPS are provided in Tables 2 and 3.

Preliminary tests aiming to verify the homogeneity and stability of the different samples were successfully performed (data not shown).

From these samples, the TPS participants were to carry out the different methods under the normal working conditions of the laboratory and in the same manner as other samples which are usually analyzed in the laboratory. PCR reagents and controls (positive controls, negative controls) were not provided by the organizer.

#### Data analysis

All statistical tests were performed using the R statistical software (version 3.3.1; R Development Core Team, Vienna, Austria). Statistical tests are considered significant for a calculated p-value lower than 5%.

### Performance criteria

#### First stage of the evaluation: Analytical specificity

In reference to the PM7/98(2) standard [4], analytical specificity (ASP) was defined as the degree of correspondence between the responses obtained by the evaluated method and the expected theoretical results (samples’ real status), and was assayed using two criteria: diagnostic sensitivity (DSE) *i*.*e*., the ability of the method to detect the target when it is present in the sample, and diagnostic specificity (DSP) *i*.*e*., the ability of the method to fail to detect the target when it is not present in the sample.

Some indeterminate results *(i*.*e*. the operator was unable to determine the status of the sample) were reported by some laboratories. Tests on the equality of the number of indeterminate results between methods on one hand and between laboratories on the other hand were performed using Fisher’s exact test and Cochran-Mantel-Haenszel’s test. The ISO 16140 standard [5] stipulates that collaborative studies should be based on data from laboratories with high competence for the techniques that are being compared. Consequently, the results of a laboratory were excluded (considered as outliers) for a given method when the statistical analysis showed a significant difference for the number of indeterminate results obtained by a laboratory compared to others and when the number of indeterminate results obtained by this laboratory represented more than 50% of indeterminate results obtained for the method and when the number of indeterminate results obtained by this laboratory represented more than 50% of expected negative or positive results obtained from the panel of samples (*i*.*e*. number of indeterminate results ≥ 5 for negative samples or ≥ 8 for positive samples). Several statistical procedures used in this paper cannot take into account missing data, so, once outliers were eliminated, we tested two scenarios: (H1) the laboratory hypothetically made the right decision for the indeterminate results in relation to the samples’ real status (*i*.*e*. the indeterminate results were counted as positive for positive sample and negative for negative samples) and (H2) the opposite. This made it possible to estimate an interval, which included the range of the parameters’ real values. However, to lighten the presentation of data, the first scenario which better reflects reality will be favored.

Total number of true positives (TP, a positive result is obtained when a positive result is expected), true negatives (TN, a negative result is obtained when a negative result is expected), false positives (FP, a positive result is obtained when a negative result is expected) and false negatives (FN, a negative result is obtained when a positive result is expected) were determined for each laboratory and each method. As explained for indeterminate results, collaborative studies should be based on data from laboratories with high competence for the techniques that are being compared. Consequently, the results of a laboratory were excluded for a given method (considered as outliers *i*.*e*. far removed from the rest of the laboratories) (i) when the expected result for at least one control was not obtained or (ii) when the number of FP or FN results obtained by this laboratory represented more than 40% of FP or FN results obtained for the method and when ≥ 50% of FP or FN results were recorded from the panel of samples (*i*.*e*. ≥ 5 FP or ≥ 8 FN).

Diagnostic specificity (DSP) was calculated using the results obtained for the negative samples (samples b, c, d, e, h, k, q, r, v) and was defined as the TN/N- ratio, where N- refers to the total number of tests for negative samples. Diagnostic sensitivity (DSE) was calculated for positive samples (samples a, f, g, i, j, l, m, n, o, p, s, t, u, w, x) and was defined as the TP/N+ ratio, where N+ refers to the total number of tests for positive samples.

Analytical specificity (ASP) was evaluated for all of the results by calculating the ratio of the sum of the number of positive and negative agreements between a method and the expected theoretical results for the number of tested samples. Confidence intervals (95%) were calculated for ASP, DSE and DSP criteria using the Wilson score method with no continuity correction [19]. Tests on the equality of ASP, DSE and DSP between methods were performed using Fisher’s exact test.

#### Second stage of the evaluation: Analytical sensitivity, repeatability, reproducibility and probability of detection model

For this second stage of the evaluation, each laboratory had to analyze 15 samples and produced 75 results. These 15 samples consisted in a serial dilution of three FD-positive DNA extracts (extracts A, B and C) in healthy grapevine DNA. There were five levels of dilution for each extract: dilution D1 = 1.0·10^−1^ (samples A1/B1/C1), dilution D2 = 1.0·10^−2^ (samples A2/B2/C2), dilution D3 = 3.3·10^−3^ (samples A3/B3/C3), dilution D4 = 1.1·10^−3^ (samples A4/B4/C4) and dilution D5 = 3.7·10^−4^ (samples A5/B5/C5). Each sample was analyzed five times.

For this second stage of the evaluation, no indeterminate results were reported by the participating laboratories. Moreover, the sub-group of five laboratories (P1, P2, P7, P12 and P14) selected for this second stage of the evaluation had already demonstrated its competence in FD detection (routine use of end-point PCR and real-time PCR protocols, and no outlier results identified during the first stage of the evaluation). Consequently, all results were included in the data analysis.

Lastly, it is worth noting that only two out of five laboratories could provide results with method Ma, whereas results were provided for all other methods by the whole sub-group of 5 laboratories. As result, for method Ma, the performance criteria were evaluated with poor precision in comparison to other methods, and for some statistical tests, no results were presented as there were insufficient data to ensure a reliable result.

According to the PM 7/76(2) standard [3], analytical sensitivity is the lowest amount of target that can be reliably detected.

For each method, a probability of detection was calculated per dilution level, according to the following equation $\frac{x}{n}$ where x corresponds to the number of positive results and n corresponds to the number of results obtained for a given dilution level. This calculated probability was compared to the theoretical detection level of 95% using the exact binomial test. For a given method, the highest dilution level (*i*.*e*. the lowest amount of target) for which no significant differences with the theoretical detection level of 95% were identified was considered as the dilution level that can be reliably detected. To have an overview for this criterion, an overall probability of detection (“overall” ASE) was calculated per method. Confidence intervals (95%) were calculated for ASE using the Wilson score method with no continuity correction [19]. Tests on the equality of ASE between methods were performed using Fisher’s exact test.

To determine variability within a laboratory and between laboratories other criteria were evaluated from the same samples and results: repeatability, reproducibility and concordance odds ratio.

Repeatability is the level of agreement between replicates of a sample tested under the same conditions according to the PM 7/76(2) standard [3]. Considering qualitative data, it was estimated by calculating accordance (DA) as recommended in the ISO 16140 standard [5] (*i*.*e*., the probability of finding the same result from two identical test portions analyzed in the same laboratory, under repeatability conditions) according to the following equation: ${\left(\frac{pr}{N}\right)}^{2}+{\left(\frac{nr}{N}\right)}^{2}$, where *pr* and *nr* are the number of positive and negative responses, respectively, and N is the total number of responses.

Reproducibility is the ability of a test to provide consistent results when applied to aliquots of the same sample tested under different conditions (time, persons, equipment, location etc.), according to the PM 7/76(2) standard [3]. Considering qualitative data, it was estimated by calculating concordance (CO) as recommended in the ISO 16140 standard [5] (*i*.*e*., the percentage chance of finding the same result for two identical samples analyzed in two different laboratories). Concordance was calculated taking each replicate in turn from each participating laboratory and pairing with the identical results from all laboratories. Concordance was the percentage of all pairings giving the same results for all possible pairings of data. If concordance is smaller than accordance, it indicates that two identical samples are more likely to give the same result if they are analyzed by the same laboratory than if they are analyzed by different ones, suggesting that there can be variability in performance between laboratories or that the method is not robust enough to reproduce the same results under different laboratory conditions. As the magnitude of qualitative repeatability and reproducibility is strongly dependent on the level of accuracy, the ISO 16140 standard [5] recommends calculating the concordance odds ratio (COR) defined as follows: $\frac{DA\;x\;(1-CO)}{CO\;x\;(1-DA)}$, to assess the degree of interlaboratory variation. The larger the odds ratio, the more predominant the interlaboratory variation. For COR values above 1.00, Fisher’s exact test was used to evaluate the statistical significance of the variation between laboratories. Confidence intervals (95%) for accordance and concordance were calculated with the basic bootstrap method [20] using R statistical software (“boot” package). To lighten the presentation of data, these confidence intervals are given only for overall results. Confidence intervals (95%) for COR values were calculated with Woolf’s logit method [21] as follows:

$$exp(ln(COR)\pm z\times \surd (\frac{1}{nDA}+\frac{1}{n(1-DA)}+\frac{1}{nCO}+\frac{1}{n(1-CO)})$$

where n is the total number of possible interlaboratory pairings for the same sample, DA is accordance, CO is concordance and z is the 1-α/2 point of the standard normal distribution (z = 1.96 for a 95% confidence interval *i*.*e*. with a risk (α) of 5%).

The approach of the PM 7/76(2) [3] and ISO 16140 [5] standards was supplemented by another approach based on the probability of detection(POD) model. This model aims to harmonize the statistical concepts and parameters between quantitative and qualitative method validation [22]. The POD model provides a tool for the graphical representation of response curves for qualitative methods. In addition, the model enables comparisons between methods, and provides calculations of repeatability, reproducibility, and laboratory effects from collaborative study data. POD characterizes the method response with respect to concentration as a continuous variable. As described previously (§ analytical sensitivity), for each method, a probability of detection was calculated per dilution level. For an interlaboratory trial, the POD value is called LPOD. Confidence intervals (95%) were calculated. The POD (or LPOD) for two methods can be compared by difference at a given analyte concentration. The statistical significance of the difference in POD values (termed dPOD or dLPOD) is determined by its confidence interval. If it includes zero, then the difference between the methods being compared is not significant.

Method variances from collaborative validation studies were modeled as follows

$$s_{R}^{2}=s_{r}^{2}+\;s_{L}^{2}$$

where s_R_ is the standard deviation of reproducibility, s_r_ is the standard deviation of repeatability, and s_L_ is the laboratory effect. The estimation method for qualitative POD variance used the analysis of variance (ANOVA) model as defined for the quantitative model given in ISO 5725–1 [23] and used the same ANOVA calculation methods, but instead of entering a qualitative result, results were coded as 1 for a positive response and 0 for a negative response.

All calculations made in this paper concerning the POD model were based on LaBuddle‘s recommendations [24] and are detailed in the publication by Wehling [22].

Variance component estimation via ANOVA with an additive model was possible because the number of replicates analyzed by each laboratory per dilution level was greater than 12 [22]).

#### Valorization of all TPS outcomes: Bayesian approach

From the results obtained by the laboratories during the first stage (to lighten the presentation of data, indeterminate results obtained during the first stage of the evaluation were considered under scenario H1 only) and the second stage of the evaluation, likelihood ratios (LR) were calculated using the following formulas:

$Positive\;LR\;(LR+)=\frac{\;SE\;}{\left(1-SP\right)}$, where SE is the proportion of positive results obtained from positive samples and SP is the proportion of negative results obtained from negative samples, *i*.*e*. the probability of a positive test result for a target sample divided by the probability of a positive result for a non-target sample.

$Negative\;LR\;(LR-)=\frac{\left(1-\;SE\right)}{SP}$, with the same notation as the previous formula, *i*.*e*. the probability of a negative test result for a target sample divided by the probability of a negative test result for a non-target sample.

The LR indicates how much a given diagnostic test result will raise or lower the pretest probability of the disease in question and is a useful tool for assessing the effectiveness of a diagnostic test [25]. The interpretation of the LRs of a diagnostic test was established as follows [25]: LRs of > 10 or < 0.1 generate large and often conclusive changes from pre- to post-test probability, LRs of 5 to 10 or 0.1 to 0.2 generate moderate shifts in pre- to post-test probability, LRs of 2 to 5 or 0.2 to 0.5 generate small (but sometimes important) changes in probability and LRs of 1 to 2 or 0.5 to 1 alter probability to a small (and rarely important) degree.

Confidence intervals (95%) for LRs were estimated using the following formula [26].

$$exp(\ln\left(\frac{p1}{p2}\right)\pm z\sqrt{\frac{\left(1-p1\right)}{p1n1}+\frac{\left(1-p2\right)}{p2n2}})$$

where for LR+, p1 = SE, p2 = 1-SP, p1n1 = true positives and p2n2 = false positives and for LR-, p1 = 1-SE, p2 = SP, p1n1 = false negatives and p2n2 = true negatives. z is the 1-α/2 point of the standard normal distribution (z = 1.96 for a 95% confidence interval).

Bayes’ theorem was further used to translate the information given by the likelihood ratios into a probability of disease. Post-test probabilities were simulated using a range (0.01%-99%) of pre-test probabilities using Bayes’ theorem as follows [27, 28]:

Pre-test probability = prevalence (defined as the proportion of grapevine plants infected by FD phytoplasma in a particular population of plants at a specific time)

$$Pre-test\;odds=\frac{prevalence}{\left(1-prevalence\right)}$$

Post−testodds=pre−testoddsxLR

$$Post-test\;probability=\frac{post-test\;odds}{\left(1+post-test\;odds\right)}$$

To combine the results of two methods, the post-test odds were calculated as follows [28]:

$$Post-test\;odds=pre-test\;odds\;x\;{LR}_{method1}\;x\;{LR}_{method2}.$$

## Results

### Analytical specificity

Results submitted by the different laboratories for the first stage of the evaluation are available in S5 Table and were used to calculate the rates of indeterminate results presented in Table 1 and the performance criteria presented in Table 4. All these results are commented on in the following sub-sections.

**Table 4:** Comparison of the performance criteria obtained during the collaborative study for the different methods (first stage of evaluation).

Methods	Analytical specificity ASP (%)^ ^^a^^d^	Diagnostic sensitivity DSE (%)^ ^^b^^d^	Diagnostic specificity DSP (%)^ ^^c^^d^	Significant variation between results produced by the method and theoretically expected results
M1	(H1) 89.6 BC	(H1) 89.5 BCD	(H1) 89.7 AB	Yes for all criteria (p < 0.001)
	(83.0–93.8)	(83.0–93.8)	(83.1–93.9)	
	(H2) 84.8 CDEF	(H2) 85.2 DE	(H2) 84.1 B	
	(77.5–90.0)	(78.0–90.4)	(76.8–89.5)	
M2	(H1) 87.5 BCDE	(H1) 84.4 DE	(H1) 92.6 AB	Yes for all criteria
	(79.9–92.5)	(76.4–90.1)	(86.1–96.2)	(H1) p < 0.001 for ASP and DSE, p = 0.006 for DSP
	(H2) 83.7 DEF	(H2) 80.0 E	(H2) 89.8 AB	(H2) p < 0.001 for all criteria
	(75.6–89.5)	(71.5–86.5)	(82.7–94.2)	
Ma	(H1) 88.3 BCDE	(H1) 84.0 DE	(H1) 95.6 AB	No for DSP ((H1) p = 0.494, (H2) p = 0.242),
	(75.8–94.8)	(70.7–91.9)	(85.2–98.8)	yes for ASP and DSE (p < 0.001)
	(H2) 85.8 BCDEF	(H2) 81.3 DE	(H2) 93.3 AB	
	(72.8–93.2)	(67.7–90.1)	(82.1–97.7)	
M3	(H1) 79.8 EF	(H1) 86.7 CDE	(H1) 68.3 CD	Yes for all criteria (p < 0.001)
	(68.3–87.8)	(76.1–93.0)	(56.0–78.4)	
	(H2) 77.4 F	(H2) 86.7 CDE	(H2) 61.9 D	
	(65.7–86.0)	(76.1–93.0)	(49.6–72.9)	
M4	(H1) 95.8 A	(H1) 96.7 A	(H1) 94.4 A	(H1) No for DSE (p = 0.060) and DSP (p = 0.059),
	(89.5–98.4)	(90.7–98.9)	(87.6–97.6)	yes for ASP (p = 0.002)
	(H2) 92.5 AB	(H2) 94.7 AB	(H2) 88.9 AB	(H2) Yes for all criteria (p = 0.007 for DSE, p = 0.002 for DSP
	(85.2–96.4)	(87.9–97.7)	(80.7–93.9)	and p < 0.001 for ASP)
M5	(H1) 91.7 AB	(H1) 93.3 ABC	(H1) 88.9 AB	Yes for all criteria
	(82.8–96.3)	(84.4–97.3)	(78.8–94.5)	(H1) p = 0.014 for DSE, p = 0.013 for DSP and p < 0.001 for ASP
	(H2) 88.7 BCD	(H2) 91.4 ABCD	(H2) 84.1 BC	(H2) p = 0.003 for DSE, p = 0.001 for DSP and p < 0.001 for ASP
	(78.6–94.4)	(81.9–96.2)	(73.2–91.1)	
M6	(H1) 95.8 A	(H1) 96.3 A	(H1) 95.1 A	(H1) No for DSE (p = 0.060) and DSP (p = 0.120),
	(89.0–98.5)	(89.7–98.7)	(88.0–98.1)	yes for ASP (p = 0.004)
	(H2) 89.4 BCD	(H2) 92.6 ABC	(H2) 84 BC	(H2) Yes for all criteria (p = 0.002 for DSE, p = 0.001 for DSP
	(80.8–94.4)	(84.8–96.6)	(74.5–90.4)	and p < 0.001 for ASP)

^a^ Analytical specificity (95% confidence interval): ability of the method to detect the target when it is present in the sample and to fail to detect the target when it is not present in the sample. Values followed by the same letter in a column are not significantly different (p = 0.05) according to Fisher’s exact test (two tailed).

^b^ Diagnostic sensitivity (95% confidence interval): ability of the method to detect the target when it is present in the sample. Values followed by the same letter in a column are not significantly different (p = 0.05) according to Fisher’s exact test (two tailed).

^c^ Diagnostic specificity (95% confidence interval): ability of the method to fail to detect the target when it is not present in the sample. Values followed by the same letter in a column are not significantly different (p = 0.05) according to Fisher’s exact test (two tailed).

^d^ For each criterion, we present data derived from the two scenarios described in the Materials & Methods section for the interpretation of indeterminate results.

#### Indeterminate results

According to the method, the rate of indeterminate results (Table 1) ranged from 2.08% (method Ma) to 6.48% (method M6). Using Fisher’s exact test, no significant differences in the rate of indeterminate results were identified between methods for the overall results and also when considering only positive or negative results (p-value respectively of 0.264, 0.275 and 0.055 for overall results, positive results and negative results). The same conclusion was reached using Cochran-Mantel-Haenszel’s test from the appropriate data set (*i*.*e*. with no missing values): p-value = 0.141 for the comparison of indeterminate results obtained with methods M1, M2, Ma, M3, M4, M5 and M6 (from the data set of laboratories P1, P2 and P9), p-value = 0.144 for the comparison of indeterminate results obtained with methods M1, M2, M3, M4, M5 and M6 (from the dataset of laboratories P1, P2, P7, P9 and P14) and p-value = 0.345 for the comparison of indeterminate results obtained with methods M1, M2, M4, M5 and M6 (from the dataset of laboratories P1, P2, P5, P7, P9, P13 and P14).

On the contrary, significant differences in the rate of indeterminate results were identified between laboratories for the overall results and also if when considering only positive or negative results (p-value respectively of 3.9·10–3, 3.4·10–4 and 1.7·10–3 for overall results, positive results and negative results). The same conclusion was reached using Cochran-Mantel-Haenzel’s: the p-value became significant (0.038) between laboratories when method M5 was introduced in the calculation, which was due to the number of indeterminate results produced by laboratory P9 with method M5. More than 50% of indeterminate results obtained for this method were recorded from this laboratory (6/11). In addition, all indeterminate results produced by laboratory P9 were obtained from negative samples, so indeterminate results represented more than 50% of expected negative results (6/9). Consequently, the results of laboratory P9 for method M5 were excluded from the analysis. From a technical point of view, it appeared that laboratory P9 was not able to determine the cut-off value for method M5 and some late Ct values were observed in two of the wells for the samples declared as indeterminate.

#### Outlier results

Expected results were obtained for all positive and negative controls in each laboratory for each method. Thus, no data were excluded from the statistical analysis for this reason.

Regardless of the indeterminate results counted (scenario H1 or H2), the results of laboratory P6 for method M2 (FP = 9 which represented, according to the scenario for indeterminate results, 45% to 53% of the number of FPs for the method) and the results of laboratory P5 for method Ma (FP = 5 which represented, according to the scenario for indeterminate results, 63% to 71% of the number of FPs for the method) should be excluded from the analysis.

The results of laboratory P9 for method M5 should also be excluded from the analysis but only for scenario H2 (PD = 7, which represents 41% of the number of FPs for the method). This result is due to the fact that this laboratory concentrated a great number of indeterminate results.

Consequently, the results of laboratory P6 for method M2, the results of laboratory P5 for method Ma and the results of laboratory P9 for method M5 were excluded from the analysis. If no clear technical explanation was found for the results of laboratory P6 for method M2, and for the results of laboratory P9 for method M5 (possible contaminations of samples during the analyses), laboratory P5 did not implement the RFLP analysis recommended for method Ma.

#### Diagnostic sensitivity and diagnostic specificity: The two components of analytical specificity

The performance criteria assessed in the first stage of the method evaluation (analytical specificity, diagnostic sensitivity and diagnostic specificity) are summarized in Table 4. The best overall performance was obtained with methods M4 and M6.

Under the two hypotheses presented in the Materials and Methods section, analytical specificity ranged from 92.5% (scenario H2) to 95.8% (scenario H1) for M4 and from 89.4% (H2) to 95.8% (H1) for M6. Using Fisher’s exact test, ASP results for methods M4 and M6 under scenario H1 were not significantly different from the results for method M5, but were significantly better than results obtained with methods M1, Ma, M2 and M3 under this same scenario.

Diagnostic sensitivity ranged from 94.7% (scenario H2) to 96.7% (scenario H1) for method M4 and from 92.6% (H2) to 96.3% (H1) for method M6. Using Fisher’s exact test, DSE results for methods M4 and M6 under scenario H1 were not significantly different from the results for method M5, but were significantly better than the results obtained with methods M1, M3, M2 and Ma under this same scenario.

Moreover, only the DSE results for methods M4 and M6 under scenario H1 presented non-significant variation with the theoretically expected results (p = 0.060).

It is worth noting that many false-negative results were obtained from sample “w” (more than 25% of laboratories obtained false-negative or indeterminate results from this sample, whatever the method used, and this percentage increased to more than 50% for methods M2, Ma, M3, M4 and M6). The presence of natural inhibitors was suspected for this sample.

Diagnostic specificity ranged from 88.9% (scenario H2) to 94.4% (scenario H1) for M4 and from 84% (H2) to 95.1% (H1) for M6. Using Fisher’s exact test, the DSP results for methods M4 and M6 under scenario H1 were not significantly different from the results for methods Ma, M2, M1 and M5, but were significantly better than the results obtained with method M3 under this same scenario.

Moreover, only the DSP results for methods M4 and M6 under scenario H1 presented non-significant variation with the theoretically expected results (p = 0.059 and p = 0.120 respectively).

The low DSP performance of method M3 (ranging from 61.9% to 68.3% depending on the scenario) was due, partially, to the positive detection of the two samples “e” (Ca. P. fraxini - 16SrVII) and “r” (Western X grapevine - 16SrIII) by, respectively, four and six laboratories out of seven participants that implemented this test. Method M3 targets the 16S rDNA of phytoplasmas of the 16SrV group but this region of the genome is a conserved region for phytoplasmas. This method is nonspecific even if the primer affinity is less significant for phytoplasmas of the 16SrVII and 16SrIII groups than for phytoplasmas of 16SrV group.

### Analytical sensitivity

**Fig 1:**
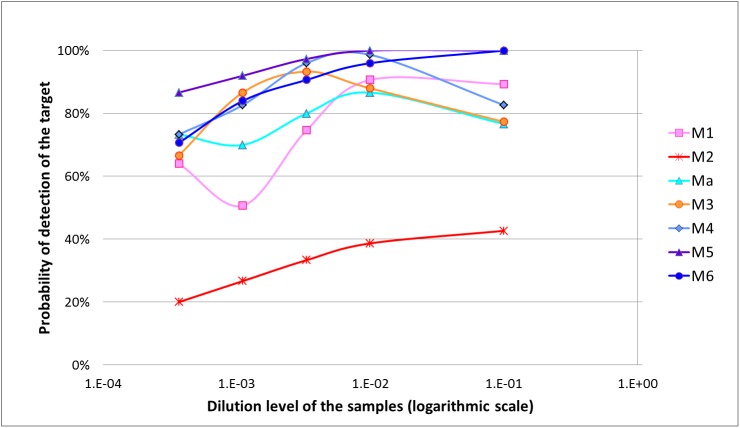
Probability of detection curves of the different methods evaluated for the detection of “Flavescence dorée” phytoplasma in plant samples.

Results submitted by the different laboratories for the second stage of the evaluation are available in S6 Table and were used to calculate the performance criteria presented in Tables 5–7 and represented in Fig 1.

**Table 5:** Results of analytical sensitivity (ASE), repeatability (DA), reproducibility (CO) and concordance odds ratio (COR) obtained during the collaborative study for the different methods.

Number of results and criteria		Results obtained for each method						
		M1	M2	Ma^a^	M3	M4	M5	M6
**Number of positive results per /number of results (per dilution level and per method) and p-value (exact binomial test) and significance with the theoretical detection level of 95%**^**c**^^**d**^	**D1**^**b**^	67/75	32/75	23/30	58/75	62/75	75/75	75/75
		(0.034 S*)	(< 0.001 S***)	(< 0.001 S***)	(< 0.001 S***)	(< 0.001 S***)	(1.000 NS)	(1.000 NS)
	**D2**	68/75	29/75	26/30	66/75	74/75	75/75	72/75
		(0.081 NS)	(< 0.001 S***)	(0.061 NS)	(0.012 S*)	(0.979 NS)	(1.000 NS)	(0.730 NS)
	**D3**	56/75	25/75	24/30	70/75	72/75	73/75	68/75
		(< 0.001 S***)	(< 0.001 S***)	(< 0.001 S***)	0.321 NS	0.730 NS	0.894 NS	0.081 NS
	**D4**	38/75	20/75	21/30	65/75	62/75	69/75	63/75
		(< 0.001 S***)	(< 0.001 S***)	(< 0.001 S***)	0.004 S**	(< 0.001 S***)	0.172 NS	(< 0.001 S***)
	**D5**	48/75	15/75	22/30	50/75	55/75	65/75	53/75
		(< 0.001 S***)	(< .001 S***)	(< 0.001 S***)	(< 0.001 S***)	(< 0.001 S***)	(0.004 S**)	(< 0.001 S***)
**Overall number of results per method**		375	375	150	375	375	375	375
**Overall number of positive results per method**		277	121	116	309	325	357	331
**Analytical sensitivity « overall ASE » (%)**^**e**^		73.9 B	32.3 C	77.3 AB	82.4 AB	86.7 AB	95.2 A	88.3 AB
		(69.2–78.1)	(27.7–37.2)	(70.0–83.3)	(78.2–85.9)	(82.9–89.7)	(92.5–96.9)	(84.6–91.1)
**Repeatability DA (%)**^**f**^		81.7	92.5	91.0	88.1	90.6	94.9	88.5
		(77.9–87.5)	(88.7–96.5)	(89.7–92.4)	(84.2–94.1)	(85.5–96.5)	(89.7–100)	(81.5–97.1)
**Reproducibility CO (%)**^**g**^		71.9	52.1	57.9	72.5	82.9	93.0	86.4
		(64.7–79.6)	(42.7–59.7)	(47.7–68.3)	(64.6–80.3)	(75.3–92.0)	(86.0–100)	(78.4–95.7)
**Odds ratio COR**^**h**^		1.74	11.41	7.36	2.79	1.99	1.39	1.22
		(1.46–2.07)	(9.18–14.18)	(4.89–11.08)	(2.30–3.39)	(1.60–2.48)	(1.03–1.88)	(0.98–1.51)
**p-value (Fisher) for significance in performance between laboratories** ^**c**^		2.06e-05 S***	4.09e-48 S***	NA^**a**^	1.54e-09 S***	8.24e-04 S***	0.08 NS	0.60 NS

^a^Values for method Ma were calculated from the results of 2 laboratories (vs 5 for other methods). Consequently, some calculations were not implemented (NA value).

^b^D1: dilution 1.0·10^−1^ (= samples A1-B1-C1); D2: dilution 1.0·10^−2^ (= samples A2-B2-C2), D3: dilution 3.3·10^−3^ (= samples A3-B3-C3); D4: dilution 1.1·10^−3^ (= samples A4-B4-C4); D5: dilution 3.7·10^−4^ (= samples A5-B5-C5)

^c^ NS: not significant (p ≥ 0.05); S*: 0.01 ≤ p < 0.05; S**: 0.001 ≤ p < 0.01; S***: p < 0.001

^d^The shaded cells indicate a statistical significance of variation with the theoretical detection level of 95%

^e^Overall analytical sensitivity (95% confidence interval): is the overall probability of detection calculated per method as the ratio between positive results and the number of results obtained per method for this second stage of the evaluation. Values followed by the same letter in a column are not significantly different (p = 0.05) according to Fisher’s exact test (two tailed)

^f^Repeatability (bootstrap confidence interval): is the probability of finding the same result from two identical test portions analyzed in the same laboratory, under repeatability conditions

^g^Reproducibility (bootstrap confidence interval): is the percentage chance of finding the same result for two identical samples analyzed in two different laboratories

^h^The concordance odds ratio (95% confidence interval) was calculated as the ratio DA x (1—CO)/CO x (1- DA). The larger the odds ratio (above 1), the more predominant interlaboratory variation

**Table 6:** Detailed results for repeatability (DA), reproducibility (CO) and concordance odds ratio (COR) obtained during the collaborative study for the different methods.

Sample codes	Criteria	Results obtained for each method						
		M1	M2	Ma^a^	M3	M4	M5	M6
**A1**	DA^**b**^	0.94	1.00	0.94	0.94	0.90	1.00	1.00
	CO^**c**^	0.68	0.40	0.20	0.68	0.76	1.00	1.00
	COR^**d**^^**f**^	7.37	inf	62.67	7.37	2.84	1.00	1.00
	CI_COR_^**e**^^**f**^	2.92–18.61	-	9.19–427.28	2.92–18.61	1.28–6.32	-	-
	P^f^^g^	0.002 S**	0.000 S***	0.048 S*	0.002 S**	0.022 S*	1.000 NS	1.000 NS
**A2**	DA	0.90	1.00	0.94	0.90	0.94	1.00	1.00
	CO	0.84	0.40	0.80	0.84	0.92	1.00	1.00
	COR	1.71	inf	3.92	1.71	1.36	1.00	1.00
	CI_COR_	0.74–3.99	-	0.57–26.71	0.74–3.99	0.45–4.08	-	-
	p	0.167 NS	0.000 S***	1.000 NS	0.167 NS	1.000 NS	1.000 NS	1.000 NS
**A3**	DA	0.94	0.90	0.94	0.94	1.00	1.00	1.00
	CO	0.92	0.52	0.80	0.92	1.00	1.00	1.00
	COR	1.36	8.31	3.92	1.36	1.00	1.00	1.00
	CI_COR_	0.45–4.08	3.88–17.80	0.57–26.71	0.45–4.08	-!	-	-
	p	1.000 NS	0.000 S***	1.000 NS	1.000 NS	1.000 NS	1.000 NS	1.000 NS
**A4**	DA	0.65	0.84	0.87	0.94	0.90	1.00	0.94
	CO	0.51	0.70	0.68	0.68	0.84	1.00	0.92
	COR	1.78	2.25	3.15	7.37	1.71	1.00	1.36
	CI_COR_	1.01–3.15	1.13–4.46	0.75–13.25	2.92–18.61	0.74–3.99	-	0.45–4.08
	p	0.290 NS	0.081 NS	1.000 NS	0.002 S**	0.167 NS	1.000 NS	1.000 NS
**A5**	DA	0.68	1.00	0.90	0.90	1.00	1.00	0.78
	CO	0.58	1.00	0.60	0.76	1.00	1.00	0.72
	COR	1.54	1.00	6.00	2.84	1.00	1.00	1.38
	CI_COR_	0.86–2.74	-	1.30–27.77	1.28–6.32	-	-	2.62
	p	0.467 NS	1.000 NS	0.444 NS	0.022 S*	1.000 NS	1.000 NS	0.753 NS
**B1**	DA	0.90	0.94	0.94	0.94	0.81	1.00	1.00
	CO	0.84	0.40	0.80	0.92	0.65	1.00	1.00
	COR	1.71	23.50	3.92	1.36	2.30	1.00	1.00
	CI_COR_	0.74–3.99	9.39–58.80	0.57–26.71	0.45–4.08	1.20–4.38	-	-
	p	0.167 NS	0.000 S***	1.000 NS	1.000 NS	0.047 S*	1.000 NS	1.000 NS
**B2**	DA	0.94	0.94	0.87	0.84	1.00	1.00	0.90
	CO	0.92	0.44	0.68	0.78	1.00	1.00	0.84
	COR	1.36	19.94	3.15	1.48	1.00	1.00	1.71
	CI_COR_	0.45–4.08	7.99–49.78	0.75–13.25	0.73–3.02	-	-	0.74–3.99
	p	1.000 NS	0.000 S***	1.000 NS	0.457 NS	1.000 NS	1.000 NS	0.167 NS
**B3**	DA	0.58	0.90	0.90	0.90	0.87	0.90	0.68
	CO	0.48	0.48	0.40	0.76	0.85	0.84	0.58
	COR	1.50	9.75	13.50	2.84	1.18	1.71	1.54
	CI_COR_	0.86–2.61	4.55–20.89	2.92–62.48	1.28–6.32	0.53–2.63	0.74–3.99	0.86–2.74
	p	0.567 NS	0.000 S***	0.167 NS	0.022 S*	1.000 NS	0.167 NS	0.467 NS
**B4**	DA	0.87	0.84	0.87	0.87	0.62	0.68	0.52
	CO	0.85	0.58	0.32	0.85	0.48	0.62	0.50
	COR	1.18	3.80	14.22	1.18	1.77	1.30	1.08
	CI_COR_	0.53–2.63	1.95–7.40	3.38–59.84	0.53–2.63	1.01–3.10	0.73–2.33	0.62–1.89
	p	1.000 NS	0.006 S**	0.206 NS	1.000 NS	0.383 NS	0.824 NS	1.000 NS
**B5**	DA	0.62	0.90	0.94	0.55	0.71	0.65	0.58
	CO	0.55	0.76	0.20	0.50	0.57	0.49	0.56
	COR	1.33	2.84	62.67	1.22	1.85	1.93	1.09
	CI_COR_	0.76–2.35	1.28–6.32	9.19–427.28	0.70–2.13	1.03–3.32	1.10–3.41	0.62–1.90
	p	0.769 NS	0.022 S*	0.048 S*	0.888 NS	0.155 NS	0.189 NS	1.000 NS
**C1**	DA	0.90	0.84	0.90	0.90	1.00	1.00	1.00
	CO	0.84	0.50	0.60	0.40	0.60	1.00	1.00
	COR	1.71	5.25	6.00	13.50	inf	1.00	1.00
	CI_COR_	0.74–3.99	2.71–10.19	1.30–27.77	6.28–29.04	-	-	-
	p	0.167 NS	0.003 S**	0.444 NS	0.000 S***	0.000 S***	1.000 NS	1.000 NS
**C2**	DA	0.81	1.00	0.94	0.94	1.00	1.00	0.94
	CO	0.71	0.40	0.80	0.68	1.00	1.00	0.92
	COR	1.74	inf	3.92	7.37	1.00	1.00	1.36
	CI_COR_	0.90–3.37	-	0.57–26.71	2.92–18.61	-	-	0.45–4.08
	p	0.160 NS	0.000 S***	1.000 NS	0.002 S**	1.000 NS	1.000 NS	1.000 NS
**C3**	DA	0.81	1.00	0.90	0.94	0.94	1.00	1.00
	CO	0.65	0.40	0.60	0.92	0.92	1.00	1.00
	COR	2.30	inf	6.00	1.36	1.36	1.00	1.00
	CI_COR_	1.20–4.38	-	1.30–27.77	0.45–4.08	0.45–4.08	-	-
	p	0.047 S*	0.000 S***	0.444 NS	1.000 NS	1.000 NS	1.000 NS	1.000 NS
**C4**	DA	0.81	1.00	0.90	0.81	1.00	1.00	1.00
	CO	0.65	0.40	0.60	0.71	1.00	1.00	1.00
	COR	2.30	inf	6.00	1.74	1.00	1.00	1.00
	CI_COR_	1.20–4.38	-	1.30–27.77	0.90–3.37	-	-	-
	p	0.047 S*	0.000 S***	0.444 NS	0.160 NS	1.000 NS	1.000 NS	1.000 NS
**C5**	DA	0.90	0.78	0.90	0.90	0.90	1.00	0.94
	CO	0.76	0.43	0.60	0.48	0.84	1.00	0.92
	COR	0.76	0.43	0.60	0.48	0.84	1.00	0.92
	CI_COR_	1.28–6.32	2.54–8.71	1.30–27.77	4.55–20.89	0.74–3.99	-	0.45–4.08
	p	0.022 S*	0.007 S**	0.444 S*	0.000 S***	0.167 NS	1.000 NS	1.000 NS

^a^Values for method Ma were calculated from the results of 2 laboratories (vs 5 for other methods).

^b^Repeatability: is the probability of finding the same result from two identical test portions analyzed in the same laboratory, under repeatability conditions

^c^Reproducibility: is the percentage chance of finding the same result for two identical samples analyzed in two different laboratories

^d^The concordance odds ratio was calculated as the ratio DA x (1—CO)/CO x (1- DA). The larger the odds ratio (above 1), the more predominant the interlaboratory variation

^e^Confidence interval (95%) of the concordance odds ratio calculated with Woolf’s logit method

^f^The shaded cells indicate statistical significance of variation between laboratories.

^g^p-value of Fisher’s exact test used to evaluate the statistical significance of the variation between laboratories: NS: not significant (p ≥ 0.05); S*: 0.01≤ p < 0.05; S**: 0.001 ≤ p < 0.01; S***: p < 0.001

**Table 7:** Statistical summary for the POD model applied to the different methods evaluated during the test performance study.

Performance criteria or parameters	Dilution levels^b^	Results obtained for each method						
		M1	M2	Ma^a^	M3	M4	M5	M6
**LPOD** ^**c**^ **(95%CI)**	D1	0.89	0.43	0.77	0.77	0.83	1.00	1.00
		(0.80; 0.94)	(0.00; 0.93)	(0.00; 1.00)	(0.59; 0.96)	(0.56; 1.00)	(0.95; 1.00)	(0.95; 1.00)
	D2	0.91	0.39	0.87	0.88	0.99	1.00	0.96
		(0.82; 0.95)	(0.00; 1.00)	(0.70; 0.95)	(0.79; 0.94)	(0.92; 1.00)	(0.95; 1.00)	(0.87; 0.99)
	D3	0.75	0.33	0.80	0.93	0.96	0.97	0.91
		(0.67; 0.82)	(0.00; 0.89)	(0.80; 0.80)	(0.85; 0.97)	(0.88; 0.99)	(0.91; 0.99)	(0.82; 0.95)
	D4	0.51	0.27	0.70	0.87	0.83	0.92	0.84
		(0.37; 0.65)	(0.00; 0.70)	(0.63; 0.77)	(0.77; 0.93)	(0.69; 0.96)	(0.84; 0.96)	(0.81; 0.87)
	D5	0.64	0.20	0.73	0.67	0.73	0.87	0.71
		(0.44; 0.84)	(0.00; 0.41)	(0.24; 1.00)	(0.47; 0.86)	(0.61; 0.85)	(0.77; 0.93)	(0.61; 0.81)
**dLPOD** ^**d**^ **(95%CI) vs. M5**^**e**^	D1	-0.11	-0.57	-0.23	-0.23	-0.17	-	0.00
		(-0.20; -0.04)	(-1 .00; -0.07)	(-1.00; 0.00)	(-0.41; -0.04)	(-0.44; 0.00)		(-0.05; 0.05)
	D2	-0.09	-0.61	-0.13	-0.12	-0.01	-	-0.04
		(-0.18;-0.03)	(-1.00; -0.00)	(-0.30;-0.04)	(-0.21; -0.05)	(-0.07; 0.04)		(-0.11; 0.02)
	D3	-0.23	-0.64	-0.17	-0.04	-0.01	-	-0.07
		(-0.31;-0.12)	(-0.97;-0.08)	(-0.19;-0.11)	(-0.12; 0.04)	(-0.09; 0.06)		(-0.16; 0.01)
	D4	-0.41	-0.65	-0.22	-0.05	-0.09	-	-0.08
		(-0.56;-0.25)	(-0.92;-0.21)	(-0.30;-0.11)	(-0.16; 0.05)	(-0.23; 0.06)		(-0.13; 0.00)
	D5	-0.23	-0.67	-0.13	-0.20	-0.13	-	-0.16
		(-0.43;-0.01)	(-0.87;-0.44)	(-0.63;0.15)	(-0.40; 0.02)	(-0.27; 0.02)		(-0.28;-0.02)
**Repeatability standard deviation (s**_**r**_**)**	D1	0.276	0.335	0.365	0.400	0.324	0.000	0.000
	D2	0.269	0.115	0.345	0.258	0.115	0.000	0.185
	D3	0.443	0.243	0.414	0.218	0.200	0.157	0.293
	D4	0.498	0.309	0.473	0.315	0.370	0.276	0.378
	D5	0.462	0.373	0.436	0.454	0.460	0.340	0.460
**Laboratory standard deviation (s**_**L**_**)**	D1	0.159	0.409	0.316	0.148	0.223	0.000	0.000
	D2	0.128	0.529	0.031	0.223	0.000	0.000	0.076
	D3	0.000	0.453	0.000	0.138	0.000	0.044	0.008
	D4	0.084	0.356	0.000	0.149	0.100	0.000	0.000
	D5	0.158	0.169	0.151	0.155	0.000	0.034	0.000
**Reproducibility standard deviation (s**_**R**_**)**	D1	0.318	0.529	0.483	0.426	0.393	0.000	0.000
	D2	0.298	0.542	0.346	0.341	0.115	0.000	0.200
	D3	0.443	0.514	0.414	0.258	0.2	0.163	0.293
	D4	0.505	0.471	0.473	0.348	0.384	0.276	0.378
	D5	0.488	0.409	0.462	0.479	0.460	0.343	0.460
**p-value of Fisher-Snedecor test (ANOVA)**^**f**^	D1	3.52e-4 S***	2.82e-12 S***	1.58e-3 S**	0.023 S*	1.881e-05 S***	-	-
	D2	3.04e-3 S***	< 2.2e-16 S***	0.299 NS	1.40e-07 S***	0.414 NS	-	0.012 S*
	D3	0.680 NS	< 2.2e-16 S***	1.000 NS	8.451e-05 S***	0.736 NS	0.083 NS	0.408 NS
	D4	0.234 NS	2.08e-11 S***	0.703 NS	3.25e-3 S***	0.091 NS	0.655 NS	0.967 NS
	D5	0.035 S*	5.00 e-3 S***	0.105 NS	0.035 S*	0.252 NS	0.342 NS	0.479 NS

^a^Values for method Ma were calculated from the results of 2 laboratories (vs 5 for other methods)

^b^D1: dilution 1.0·10^−1^ (= samples A1-B1-C1); D2: dilution 1.0·10^−2^ (= samples A2-B2-C2), D3: dilution 3.3·10^−3^ (= samples A3-B3-C3); D4: dilution 1.1·10^−3^ (= samples A4-B4-C4); D5: dilution 3.7·10^−4^ (= samples A5-B5-C5)

^c^Laboratory probability of detection values: probability of detection values obtained during an interlaboratory comparison

^d^Difference in laboratory probability of detection values

^e^Differences in LPOD values between each method and method M5 (considered in the calculation as the reference method). The shaded cells indicate a statistical significance between the compared method and M5 based on the fact that the confidence interval for dLPOD does not include zero

^f^The shaded cells indicate statistical significance for POD between laboratories. NS: not significant (p ≥ 0.05); S*: 0.01≤ p < 0.05; S**: 0.001 ≤ p < 0.01; S***: p < 0.001

Fig 1 provides a graphical representation of the probability of detection of the different methods based on the dilution level of the target. Graphically, the results of method M2 are largely below those of the other methods, whereas method M5 appears to be the best method regardless of the dilution level.

We can note that some results seem to be inconsistent with the serial dilution: method M1 (dilution D1), method Ma (D1), method M4 (D1) and method M3 (D1 and D2). If there were differences between laboratories, no evidence of outlier results could be identified (lower probability of detection could not be related to one laboratory in particular), so all data were included in the statistical analysis. The presence of natural inhibitors in the samples could explain these unexpected results at low dilution levels, just as for sample “w” in the evaluation of diagnostic sensitivity (it can be noted that sample “w” was used to produce the “C” serial dilution). The inhibition occurring at low dilutions could be removed at high dilution levels (by the dilution of inhibitors).

The analytical sensitivity results for the different methods are summarized in Table 5. Best results were obtained for method M5 for which the target could be reliably detected up to the D4 dilution (no significance with the theoretical detection level of 95% identified using the exact binomial test). For method M6, this level corresponded to the D3 dilution. For methods M4, M3 and Ma, results were less interpretable because of inconsistent results in the first dilution levels as previously described. However, method M4 presented reliable detection of the target for dilution levels D2 and D3. By contrast, results for method M2 were significantly different from the theoretical detection level of 95%, for all dilution levels. The results of overall ASE confirmed these results: using Fisher’s exact test, the overall ASE of method M2 (32.3%) was significantly lower than for other methods.

### Repeatability, reproducibility and odds ratio

Overall repeatability (Table 5) was above 90% for methods M5, M2, Ma and M4, whereas overall reproducibility was above 90% only for method M5. While repeatability remained good for all methods (greater than 80%), the results for reproducibility were very poor for some methods (52.1% and 57.9% for M2 and Ma respectively).

The concordance odds ratio was not significantly different from 1.00 only for methods M5 and M6 (Fisher’s exact test), meaning that no significant differences between laboratories were identified concerning the results obtained from these two methods. Similar results were obtained when results per sample were considered (Table 6). In the case of method M4, even though the overall COR was significantly different from 1.00, it is worth noting that when results per sample were considered, only COR results for samples corresponding to the D1 dilution were significant. No significant differences between laboratories were identified concerning the results obtained from samples corresponding to dilutions D2 to D5 for method M4. For other methods, the overall COR was significant and in detail, differences between laboratories were identified for many samples and dilution levels. Greater differences between laboratories were identified for methods M2 and Ma.

In particular, the case of method M2 is intriguing regarding the significance of the difference between laboratories: three laboratories presented an overall probability of detection close to 5% whereas the other two laboratories presented a probability of detection close to 70%. The case of method Ma, even if it should be put into perspective given the very small number of laboratories able to produce results with this method, already reflects problems in the reproducibility of results between laboratories. This is broadly confirmed from a technical point of view by the fact that more than 50% of laboratories could not provide results with this method during this second stage of the evaluation.

### Probability of detection model

Statistical parameters of the POD model applied to the different methods are shown in Table 7. For the calculations of dLPOD values, method M5 was considered as the reference method (in regard to the previous results), and side-by-side comparisons occurred between M5 and each of the other methods.

The results for repeatability standard deviation, reproducibility standard deviation and laboratory effect were consistent with previous results concerning repeatability, reproducibility and COR. The laboratory effect was particularly high for method M2 (0.169 to 0.529, depending on the dilution level), whereas it was very low (equal or close to zero) for methods M5 and M6, and for method M4 for all dilutions except for D1. The p-value associated with the Fisher-Snedecor test in the ANOVA performed from binary results obtained by each laboratory indicated a significant difference between laboratories for all dilution levels for methods M2 and M3, a significant difference for three dilution levels (D1, D2 and D5) for method M1, and a significant difference for only one dilution level for methods Ma (D1), M4 (D1) and M6 (D2). No significant differences between laboratories were identified for method M5.

Lastly, using the dLPOD for comparing the responses of methods with respect to the target concentration, no significant differences were identified between methods M4 and M5, and between methods M6 and M5 (except for the highest dilution, D5). Significant differences were identified for all dilution levels for methods M1 and M2, for three dilution levels (D2, D3 and D4) for method Ma and for two dilutions levels (D1 and D2) for method M3.

### Bayesian approach

Likelihood ratios are shown in Table 8. The LR+ values from methods M6, Ma and M4 (respectively 18.30, 17.90 and 16.11) are high (> 10), indicating that these methods generate a large change from pre- to post-test probability. The reliability of a positive test result is therefore higher for these methods than for methods M5, M1 and M2 (moderate change: LR+ values between 5 and 10) and more particularly than for M3 (small change: LR+ = 2.63 < 5). The LR- of M5 is very close to zero (equal to 0.06 < 0.1), indicating that this method generates a large change from pre- to post-test probability. The reliability of a negative test result is therefore much higher for this method than for methods M6 and M4 (moderate change: LR- values between 0.1 and 0.2) and more particularly than for methods Ma, M1, M3 and M2 (small change: LR- > 0.2).

**Table 8:** Comparison of likelihood ratios obtained during the collaborative study for the different methods.

Methods	LR+^ ^^a^^d^		LR-^ ^^b^^d^	
	Value ^c^	Change from pre-to post-probability	Value ^c^	Change from pre-to post-probability
M1	7.70	Moderate	0.23	Small
	(4.60–12.91)		(0.19–0.27)	
M2	6.64	Moderate	0.55	Small
	(3.39–13.00)		(0.50–0.61)	
Ma	17.90	Large	0.21	Small
	(4.61–69.49)		(0.16–0.28)	
M3	2.63	Small	0.24	Small
	(1.82–3.78)		(0.19–0.32)	
M4	16.11	Large	0.11	Moderate
	(6.87–37.79)		(0.09–0.14)	
M5	8.53	Moderate	0.06	Large
	(4.24–17.16)		(0.04–0.09)	
M6	18.30	Large	0.10	Moderate
	(7.04–47.61)		(0.08–0.13)	

^a^ The positive likelihood ratio LR+ (95% confidence interval) was defined as the ratio $\frac{SE}{\left(1-SP\right)}$, where SE refers to the proportion of positive results obtained from positive samples and SP refers to the proportion of negative results obtained from negative samples.

^b^ The negative likelihood ratio LR- was defined as the ratio $\frac{\left(1-SE\right)}{SP}$, where SE refers to the proportion of positive results obtained from positive samples and SP refers to the proportion of negative results obtained from negative samples

^c^ Value of likelihood ratio (95% confidence interval)

^d^ We present data derived from scenario H1 described in the Materials & Methods section for the interpretation of indeterminate results

**Fig 2:**
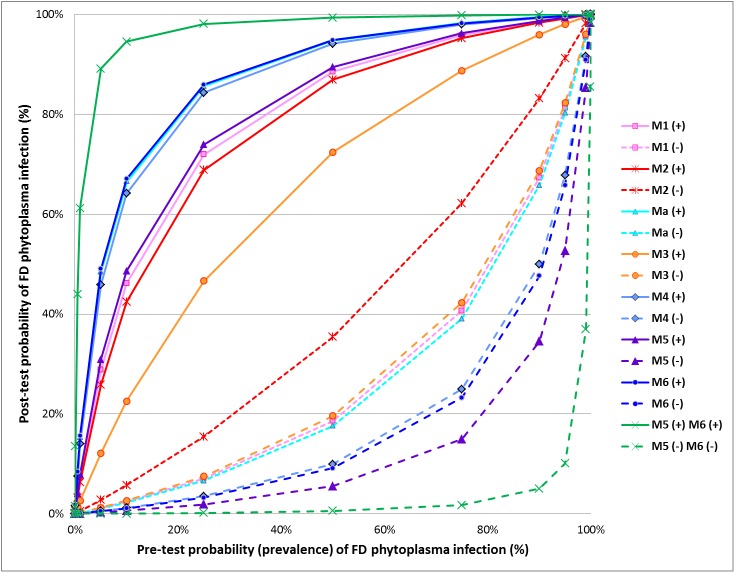
Relationship between pre- and post-test probabilities of “Flavescence dorée” phytoplasma infection, according to the results obtained during the interlaboratory test performance study for each evaluated method and for the combination of both methods M5 and M6.

The likelihood ratio can be combined with the prevalence of infection to determine the post-test probability of infection. Fig 2 illustrates the post-test probabilities of FD phytoplasma (*i*.*e*. after a test result) as a function of the pre-test probabilities for each evaluated method and also for the combination of the two most reliable methods (methods M5 and M6). In this graph, the effect of the test result is described by two curves, one for a positive result and the other for a negative one (Lamb, 2007), making it possible to calculate the post-test probability of infection with a positive or negative result depending on the prevalence of the FD phytoplasma in the studied population. For example, in a population with a prevalence of 50%, the probability of a tested individual really being infected after a positive result is higher than 90% for methods Ma, M6 and M4; it is between 80% and 90% for methods M5, M1 and M2 and only 73.2% for M3. After a negative result, there is only 5.5% probability that the grapevine plant is infected by the FD phytoplasma when tested with method M5. This probability increases to 9.2% and 10.0% for methods M6 and M4 respectively, but remains low for these methods. Conversely, relatively high probabilities of infection are found for samples tested negative with Ma, M1, M3 and particularly M2 (35.4%).

The Bayesian approach can be used to choose the most appropriate detection scheme for a particular epidemiological situation. If disease prevalence is low, as is usually the case for FD phytoplasma detection, one of the three real-time PCR methods (M4, M5 or M6) is the most convenient test for routine FD phytoplasma assessment. However, when the FD phytoplasma-free status needs to be accurately assessed (*e*.*g*. for the production of healthy plants and the grapevine certification scheme), it would be appropriate to use two detection tests both based on real-time PCR (*e*.*g*. methods M5 and M6). The probability of infection of a plant with a positive result obtained with two detection tests is higher than the probability of infection of a plant with a positive result obtained by only one detection test. Similarly, the accuracy of a negative result is very high when the analysis is performed by both detection tests. For example, the post-test probability of infection is lower than 1% if a negative result is obtained both with method M5 and method M6 from a grapevine plant sampled in a population presenting up to 63% prevalence of infection (vs. 14% if method M5 is used alone). This suggests that the combination of methods could minimize the risk of releasing infected material when the two test results are negative, which is particularly important for the certification of grapevine plants. Similarly, the post-test probability of infection is higher than 90% with a positive result obtained both with method M5 and method M6 from a grapevine plant sampled in a population with at least 6% prevalence (vs. at least 30% prevalence if method M6 is used alone). It can be important to guarantee a positive result through the use of two tests if grubbing up and destruction decisions with major economic consequences are taken on the basis of these analysis results.

Pre-test probability (prevalence) was defined as the proportion of plants infected by FD phytoplasma in a particular population at a specific time. Post-test probability was calculated as follows: $\frac{post-test\;odds}{\left(1\;+\;post-test\;odds\right)}$, where $post-test\;odds=\frac{\;pre-test\;probability}{(1\;–pre-test\;probability)\;}x\;likelihood\;ratio$. For each method, the solid line represents the post-test probabilities of FD phytoplasma infection after a positive test result for different prevalence rates. The broken line represents the post-test probabilities of FD phytoplasma infection after a negative test result for different prevalence rates.

## Discussion

**Fig 3:**
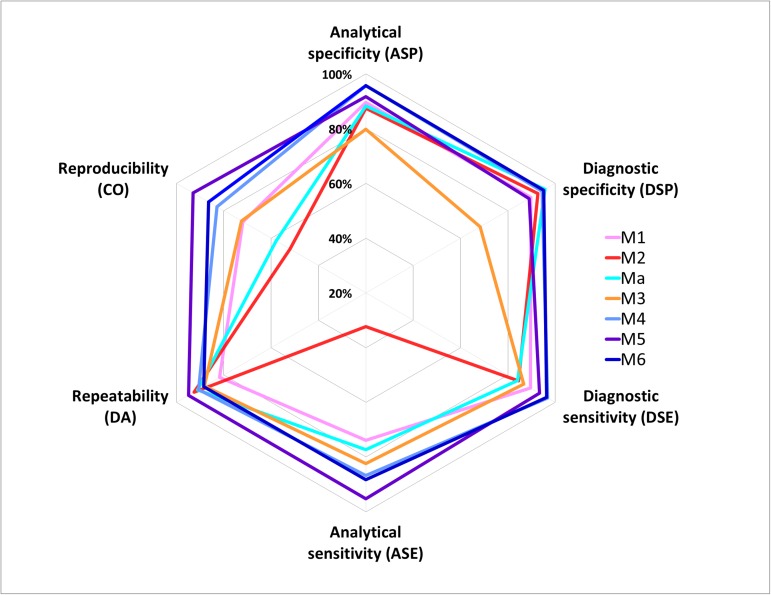
Diagram summarizing the performance of the different methods evaluated in the interlaboratory test performance study.

This interlaboratory test performance study showed that real-time PCR protocols developed by Hren [16] (M4), Pelletier [17] (M5) and under-patent oligonucleotides (M6) achieved the highest levels of performance for FD phytoplasma detection (Fig 3).

During the first stage of the evaluation based on the analysis of a variety of target and non-target samples for the determination of analytical specificity, the best results were obtained with methods M4 and M6. The results obtained with methods M4 and M6 were not significantly different from the results of M5, but were significantly better than the results obtained with other methods.

During the second stage of the evaluation based on the analysis of serial dilutions of target samples for the determination of analytical sensitivity, repeatability, reproducibility, and the POD model, the best results were obtained with method M5 for which the target can be reliably detected up to the dilution of 1.1·10^−3^, with overall repeatability and reproducibility higher than 90%. The concordance odds ratio was not significantly different from 1.00 only for methods M5 and M6, meaning that no significant differences between laboratories were identified concerning the results obtained from these two methods, whereas significant differences were identified for all other methods (however for M4 only for the first dilution level). Using the POD model, no significant differences were identified between M4 and M5, or between M6 and M5 (except for the highest dilution, 3.7·10^−4^). Significant differences were identified for at least two dilution levels (and for up to all dilution levels) for other methods.

Lastly, the Bayesian approach helps summarize all these results and choose the most appropriate detection scheme depending on the epidemiological context. The graphical representation of post-test probabilities of FD phytoplasma as a function of pre-test probabilities for each evaluated method demonstrate the relevance of methods M4 and M6 for the positive predictive value (*i*.*e*. confidence in the positive test result), and the relevance of method M5 for the negative predictive value (*i*.*e*. confidence in the negative test result). The combination of methods M5 and M6 minimizes the risk of releasing infected material when the two test results are negative, which is particularly important for the certification of grapevine plants. Similarly, it can be important to guarantee a positive result through the use of two tests if grubbing up and destruction decisions with major economic consequences are taken on the basis of these analysis results.

This paper underlines the usefulness of interlaboratory trials for method validation. These studies are essential to establish the reliability and compatibility of test results. They enable the evaluation of performance criteria such as reproducibility that are essential to evaluate how transferable the method is among laboratories performing routine analyses. For plant pathology, collaborative studies remain rare [29], although they are recommended by different regional and international organizations in plant health such as the European Plant Protection Organization.

The use of statistics in the data processing of interlaboratory collaborative studies is essential to identify significant differences in performance criteria between tests *i*.*e*. to ensure that the differences observed between tests have a high probability of being real and not due to chance.

However the situation can be more complex. For example, when comparing two methods, we can come to the conclusion that the first method shows better performance than the second one for a criterion (e.g. analytical specificity) but poorer performance for another criterion (e.g. analytical sensitivity). Thus, in this type of situation, it is very difficult to draw a conclusion as to the overall performance of the two methods. This case precisely demonstrates the relevance of new statistical approaches such as the probability of detection model and the Bayesian approach. Supplementing the traditional approach, these new statistical approaches provide an overview of method performance. They are essential tools to reliably compare methods in their entirety (including the diversity of contamination levels, the diversity of the target, the diversity of sample matrices, etc.). Graphical representations are used to summarize and communicate the results. In the case of the POD model, the graph (Fig 1) summarizes the rate of positive results as a function of the target concentration. Anyone can see at a glance that methods M5 and M6 have the best performance overall when considering different contamination levels; however it remains important to refer to the calculated values (e.g. Table 7) to evaluate the significance of these differences. In the case of the Bayesian approach, the graph (Fig 2) summarizes method performance, including both the results for positive and negative samples and simulating different epidemiological situations. It is very useful to quickly identify the most appropriate methods according to the epidemiological context. For example, in situations of high prevalence, method M5 appears as the most appropriate method to confirm a negative result, whereas in situations of low prevalence, methods M6, M5 and Ma appear as the most appropriate methods to confirm a positive result. This graph also provides users with an overview of method performance irrespective of the prevalence and the type of sample (positive or negative). For a given method, the closer to the vertical and horizontal axes the solid (and respectively the dotted) curves are, the higher the overall method performance is. Thus, it is easy to identify methods M4, M5 and M6 as being the most effective in all situations. Lastly, the graph illustrates the relevance of combining two different methods (e.g. M5 and M6).

The contribution of the POD model and Bayesian approaches for qualitative methods can be compared to that of the accuracy profile which is now widely used for quantitative analytical methods [30, 31].However, this paper also illustrates the difficulty in plant pathology of designing a TPS perfectly in line with theoretical statistical rules. Current available guidelines recommend a minimum of ten valid laboratory data sets (and not fewer than eight). This number can be difficult to meet in plant pathology for several reasons including the lack of availability of reference materials and the small number of laboratories competent for a given pathogen. Consequently, very often interlaboratory test performance studies are driven by collaborative projects such as EUPHRESCO which involve different partners at a regional or an international scale (European scale for EUPHRESCO), making it possible to meet the required number of competent laboratories. However, the downside is that these TPSs usually include a variety of methods to be evaluated, each partner legitimately wishing to include the method it routinely uses in the TPS. Thus, the need for a consensus in collaborative projects generally leads to a wide variety of methods being included, making it difficult for all the participants to implement all the methods.

For example, in the case of TPS for FD phytoplasma detection, for the first step of the evaluation, methods were implemented by five to 14 laboratories. This created distortion in the precision of assessment of the methods. In this paper, we have made the decision to process all the data mentioning this distortion of precision with all the caveats, and being aware that the non-significance of a statistical test does not mean the absence of differences, but rather the non-identification of differences.

This also raises the question of the competence of laboratories participating in a TPS. The more methods in the TPS, the more laboratories may be led to implement methods with which they are not familiar. Hence the importance of establishing upstream criteria to identify outliers. The issue of indeterminate results is also important to consider because the impact can be very different from one method to another. Not to integrating indeterminate results in calculations can lead to bias in the results. In this paper, significant differences in the number of indeterminate results were identified between laboratories but not between methods, and therefore the impact of the presence of indeterminate results on the method assessment was low. A number of questions we need to deal with in the data processing stage should be anticipated in the design of TPS issues. When designing a TPS, special effort should be made upstream to define how data will be used and processed and to consequently define the appropriate experimental design (number of participants, number of evaluated methods in line with the number of participants, number of target and non-target samples, number of repetitions, number of dilution (or concentration) levels, diversity/representativeness of samples etc.). However, this need for design framing should not be a pretext to include only artificially contaminated samples. This collaborative study, with the example of sample “w” underlines the fact that it is essential to include in the sample selection, when possible, naturally contaminated samples, which match as closely as practicable the type of samples encountered in routine testing, to assess the tests in “real” conditions.

In conclusion, this paper underlines the usefulness of statistics to increase the reliability of validation data. The statistical tools presented in this paper, some standard and some new in plant health, are deliberately exhaustive to provide useful guidelines for research staff wanting to validate new detection tests through interlaboratory collaborative studies. They can be applied to many other studies concerning plant pathogens and other disciplines that use qualitative detection methods.

## Supporting information

S1 Table List of participants in the interlaboratory test performance study. (DOC)

S2 Table Amplification conditions of conventional PCR methods evaluated during the interlaboratory test performance study concerning Flavescence dorée (FD) detection (DOCX)

S3 Table Amplification conditions of real-time PCR methods evaluated during the interlaboratory test performance study concerning Flavescence dorée (FD) detection (DOCX)

S4 Table Method implemented by each participant at each stage of the evaluation. (DOC)

S5 Table Results submitted by the different laboratories during the collaborative study for the first stage of the evaluation. (DOC)

S6 Table Results submitted by the different laboratories during the collaborative study for the second stage of the evaluation. (DOC)
