# Supplementary material for: Data processing of qualitative results from an interlaboratory comparison for the detection of “Flavescence dorée” phytoplasma: How the use of statistics can improve the reliability of the method validation process in plant pathology
Source: PLoS One. 2017 Apr 6;12(4):e0175247. doi: 10.1371/journal.pone.0175247 (PMC5383269; doi:10.1371/journal.pone.0175247)
Supplement: S1 Table — (DOC) [file pone.0175247.s001.doc]

**TABLE S1.** List of participants in the interlaboratory test performance study.

| Laboratory | Country | Town |
| --- | --- | --- |
| AT-AGES  Austrian Agency for Health and Food Safety | Austria | Wein |
| BE-CRA-W  Walloon Agricultural Research Centre | Belgium | Gembloux |
| BE-ILVO  Institute for Agricultural and Fisheries Research | Belgium | Merelbeke |
| CH-ACW  Agroscope Changins-Wädenswil | Switzerland | Nyon |
| ES-IRTA  Institut de Recerca i Tecnologia Agroalimentaries | Spain | Barcelona |
| FR-ANSES^a^  Plant Health Laboratory | France | Angers |
| IT-CRA-PAV  Plant Pathology Research Centre | Italy | Rome |
| IT-CRA-VIT  Centro di Ricerca per la Viticoltura | Italy | Susegana |
| IT-DipSA  *Alma Mater Studiorum*, University of Bologna | Italy | Bologna |
| IT-DISAA  Dipartimento di Produzione Vegetale, University of Milan | Italy | Milan |
| PT-INIAV  National Institute of Agrarian and Veterinary Research | Portugal | Oeiras |
| RS-IPEP  Laboratory of Applied Phytopathology  Institute of Pesticides and Environmental Protection | Serbia | Belgrade |
| SI-NIB  National Institute of Biology  Department of plant physiology and biotechnology | Slovenia | Ljubljana |
| TR-PPRS  Plant Protection Research Station | Turkey | Bornova |

^a^Coordination and organization of the interlaboratory test performance study
