# Supplementary material for: Data processing of qualitative results from an interlaboratory comparison for the detection of “Flavescence dorée” phytoplasma: How the use of statistics can improve the reliability of the method validation process in plant pathology
Source: PLoS One. 2017 Apr 6;12(4):e0175247. doi: 10.1371/journal.pone.0175247 (PMC5383269; doi:10.1371/journal.pone.0175247)
Supplement: S2 Table — (DOCX) [file pone.0175247.s002.docx]

**TABLE S2.** Amplification conditions of conventional PCR methods evaluated during the interlaboratory test performance study concerning Flavescence dorée (FD) detection

| Method | Amplification step | Primer pairs for FD detection | Concentrations | | | | Amplification [time(s)/T(°C)] | | | | | |
| --- | --- | --- | --- | --- | --- | --- | --- | --- | --- | --- | --- | --- |
|  |  |  | Pol^a^  (U) | Primers (nM) | dNTP (µM) | MgCl_2_ (mM)^b^ | ID^c^ | Amplification cycle | | | N^d^ | FE^e^ |
| M1 | First amplification | P1/P7 | 0.625 | 400 | 200 |  | 120/94 | 60/94 | 60/55 | 120/72 | 35 | 600/72 |
|  | Nested PCR | R16(V)F1/R16(V)R1 | 0.625 | 400 | 200 |  | 120/94 | 60/94 | 60/50 | 120/72 | 35 | 600/72 |
| M2 | First amplification | FD9f1/FD9r1 | 0.6 | 340 | 136 | 1.93 | 180/92 | 60/92 | 60/55 | 90/72 | 35 | 600/72 |
|  | Nested PCR | FD9f3b/FD9r2 | 0.6 | 300 | 136 | 1.93 | 180/92 | 60/92 | 60/56 | 90/72 | 35 | 600/72 |
| Ma | First amplification | P1/P7 | 0.625 | 400 | 200 |  | 120/95 | 60/94 | 60/55 | 180/72 | 35 | 600/72 |
|  | Nested PCR | M1/B6 | 0.625 | 400 | 200 |  | 120/95 | 60/94 | 60/50 | 180/72 | 35 | 600/72 |

^a^ For M1 and Ma, the recommended DNA polymerase was GoTaq^®^ DNA polymerase (Promega) but the participants were free to use another one. For M2, no DNA polymerase was recommended; participants were free to use the enzyme they usually use.

^b^ The 5X buffer of the GoTaq^®^ DNA polymerase (Promega) already contains 7.5mM of MgCl_2_ and it was used at 1X.

^c^ Initial denaturation

^d^ Number of cycles

^e^ Final elongation
