# Supplementary material for: Data processing of qualitative results from an interlaboratory comparison for the detection of “Flavescence dorée” phytoplasma: How the use of statistics can improve the reliability of the method validation process in plant pathology
Source: PLoS One. 2017 Apr 6;12(4):e0175247. doi: 10.1371/journal.pone.0175247 (PMC5383269; doi:10.1371/journal.pone.0175247)
Supplement: S3 Table — (DOCX) [file pone.0175247.s003.docx]

**TABLE S3.** Amplification conditions of real-time PCR methods evaluated during the interlaboratory test performance study concerning Flavescence dorée (FD) detection

| Method | Primer pair for FD detection | Probe | Recommended master mix^a^ | Concentrations | | | Amplification [time(s)/T(°C)] | | | | |
| --- | --- | --- | --- | --- | --- | --- | --- | --- | --- | --- | --- |
|  |  |  |  | Master mix (X) | Primers  (nM) | Probe (nM) | UDG AS^b^ | ID^c^ | Amplification cycle | | N^d^ |
| M3 | Flavescence dorée forward/reverse | Flavescence dorée probe | Platinum^®^ Quantitative PCR SuperMix-UDG (Invitrogen^TM^) | 1 | 200 | 150 | 180/50 | 180/95 | 15/95 | 60/60 | 50 |
| M4 | FDgen forward/reverse | FDgen probe | TaqMan^®^ Universal PCR Master Mix (Applied Biosystems^TM^) | 1 | 900 | 250 | 120/50 | 600/95 | 15/95 | 60/60 | 45 |
| M5 | mapFD-F/mapFD-R | mapFD-FAM | Master mix adapted for multiplex real-time PCR | 1 | 200 | 200 |  | 900/95 | 60/94 | 90/59 | 45 |
| M6 | F2263/R2362 | FD66 | iQ^TM^ Multiplex Powermix (Bio-Rad) | 1 | 400 | 150 |  | 600/95 | 15/95 | 50/60 | 45 |

^a^ These master mixes were recommended but the participants were free to use other ones

^b^ UDG activation step, only if UDG present.

^c^ Initial denaturation

^d^ Number of cycles
