# Supplementary material for: Data processing of qualitative results from an interlaboratory comparison for the detection of “Flavescence dorée” phytoplasma: How the use of statistics can improve the reliability of the method validation process in plant pathology
Source: PLoS One. 2017 Apr 6;12(4):e0175247. doi: 10.1371/journal.pone.0175247 (PMC5383269; doi:10.1371/journal.pone.0175247)
Supplement: S4 Table — (DOC) [file pone.0175247.s004.doc]

**TABLE S4.** Method implemented by each participant at each stage of the evaluation.

| **Stage of the evaluation** | **Laboratory** | **Method 1** | **Method 2** | **Method a** | **Method 3** | **Method 4** | **Method 5** | **Method 6** |
| --- | --- | --- | --- | --- | --- | --- | --- | --- |
| **Evaluation of analytical specificity** | P1 | X | X | X | X | X | X | X |
|  | P2 | X | X | X | X | X | X | X |
|  | P3 | X | X |  |  | X |  |  |
|  | P4 | X | X |  |  |  |  |  |
|  | P5 | X | X | X^a^ |  | X | X | X |
|  | P6 | X | X^a^ |  |  |  |  |  |
|  | P7 | X | X |  | X | X | X | X |
|  | P8 | X | X | X |  |  |  |  |
|  | P9 | X | X | X | X | X | X^a^ | X |
|  | P11 | X | X | X |  |  |  |  |
|  | P12 | X | X |  | X | X | X | X |
|  | P13 | X | X |  |  | X | X | X |
|  | P14 | X | X |  | X | X | X | X |
|  | P15 | X |  |  | X | X |  | X |
| **Evaluation of analytical sensitivity,** **repeatability and reproducibility** | P1 | X | X | X | X | X | X | X |
|  | P2 | X | X | X | X | X | X | X |
|  | P7 | X | X |  | X | X | X | X |
|  | P12 | X | X |  | X | X | X | X |
|  | P14 | X | X |  | X | X | X | X |

^a^Data excluded from the analysis
