# Supplementary material for: Data processing of qualitative results from an interlaboratory comparison for the detection of “Flavescence dorée” phytoplasma: How the use of statistics can improve the reliability of the method validation process in plant pathology
Source: PLoS One. 2017 Apr 6;12(4):e0175247. doi: 10.1371/journal.pone.0175247 (PMC5383269; doi:10.1371/journal.pone.0175247)
Supplement: S5 Table — (DOC) [file pone.0175247.s005.doc]

*TABLE S5. Results submitted by the different laboratories during the collaborative study for the first stage of the evaluation.*

| **Method** | **Sample code** | **Expected results^a^** | **Results from the different laboratories^ab^** | | | | | | | | | | | | | |
| --- | --- | --- | --- | --- | --- | --- | --- | --- | --- | --- | --- | --- | --- | --- | --- | --- |
|  |  |  | **P1** | **P2** | **P3** | **P4** | **P5** | **P6** | **P7** | **P8** | **P9** | **P11** | **P12** | **P13** | **P14** | **P15** |
| **Method 1** | **a** | **1** | 1 | 1 | 1 | 1 | 1 | 1 | 1 | 1 | 1 | 1 | 1 | 1 | 1 | 1 |
|  | **b** | **0** | 0 | 0 | 0 | 2 | 0 | 0 | 1 | 0 | 0 | 0 | 0 | 0 | 0 | 0 |
|  | **c** | **0** | 0 | 0 | 0 | 0 | 0 | 0 | 0 | 0 | 0 | 0 | 0 | 1 | 0 | 0 |
|  | **d** | **0** | 0 | 0 | 0 | 0 | 0 | 0 | 0 | 0 | 2 | 0 | 0 | 0 | 0 | 0 |
|  | **e** | **0** | 1^b^ | 0 | 1 | 0 | 2 | 0 | 0 | 0 | 1 | 1 | 1 | 0 | 2 | 0 |
|  | **f** | **1** | 1 | 1 | 1 | 0 | 1 | 1 | 2 | 1 | 1 | 1 | 1 | 1 | 1 | 1 |
|  | **g** | **1** | 1 | 1 | 1 | 1 | 1 | 0 | 2 | 1 | 1 | 1 | 1 | 1 | 1 | 1 |
|  | **h** | **0** | 0 | 0 | 2 | 0 | 0 | 0 | 1 | 0 | 0 | 0 | 0 | 0 | 0 | 0 |
|  | **i** | **1** | 1 | 1 | 1 | 0 | 1 | 0 | 1 | 1 | 1 | 1 | 1 | 1 | 0 | 1 |
|  | **j** | **1** | 1 | 1 | 1 | 1 | 1 | 1 | 1 | 1 | 1 | 1 | 1 | 1 | 2 | 1 |
|  | **k** | **0** | 0 | 0 | 0 | 0 | 0 | 0 | 1 | 0 | 0 | 0 | 0 | 1 | 0 | 0 |
|  | **l** | **1** | 1 | 1 | 1 | 0 | 1 | 0 | 1 | 1 | 1 | 1 | 1 | 1 | 1 | 1 |
|  | **m** | **1** | 1 | 1 | 1 | 1 | 1 | 1 | 1 | 1 | 1 | 1 | 1 | 1 | 1 | 1 |
|  | **n** | **1** | 1 | 1 | 1 | 2 | 1 | 1 | 1 | 1 | 1 | 1 | 1 | 1 | 1 | 1 |
|  | **o** | **1** | 1 | 1 | 1 | 0 | 1 | 0 | 1 | 1 | 1 | 1 | 1 | 1 | 1 | 1 |
|  | **p** | **1** | 1 | 0 | 1 | 1 | 1 | 1 | 1 | 0 | 1 | 0 | 1 | 1 | 0 | 1 |
|  | **q** | **0** | 0 | 0 | 0 | 0 | 0 | 0 | 1 | 0 | 0 | 0 | 0 | 1 | 0 | 0 |
|  | **r** | **0** | 0 | 0 | 0 | 0 | 0 | 0 | 1 | 0 | 0 | 0 | 0 | 0 | 0 | 0 |
|  | **s** | **1** | 2 | 1 | 1 | 0 | 1 | 1 | 1 | 1 | 1 | 1 | 1 | 0 | 1 | 1 |
|  | **t** | **1** | 1 | 1 | 1 | 1 | 1 | 1 | 2 | 1 | 1 | 1 | 1 | 1 | 1 | 1 |
|  | **u** | **1** | 1 | 1 | 1 | 1 | 1 | 1 | 0 | 1 | 1 | 1 | 1 | 1 | 1 | 1 |
|  | **v** | **0** | 0 | 2 | 0 | 0 | 0 | 0 | 0 | 0 | 0 | 0 | 0 | 2 | 0 | 0 |
|  | **w** | **1** | 1 | 2 | 1 | 1 | 1 | 1 | 0 | 1 | 1 | 1 | 1 | 0 | 1 | 0 |
|  | **x** | **1** | 1 | 2 | 1 | 0 | 1 | 0 | 0 | 1 | 1 | 1 | 1 | 1 | 2 | 1 |
|  |  |  | **P1** | **P2** | **P3** | **P4** | **P5** | **P6**^c^ | **P7** | **P8** | **P9** | **P11** | **P12** | **P13** | **P14** | **P15** |
| **Method 2** | **a** | **1** | 1 | 0 | 1 | 1 | 1 | 1 | 1 | 0 | 1 | 2 | 1 | 1 | 1 |  |
|  | **b** | **0** | 0 | 0 | 0 | 0 | 0 | 1 | 1 | 0 | 0 | 0 | 0 | 0 | 0 |  |
|  | **c** | **0** | 0 | 0 | 0 | 0 | 0 | 1 | 1 | 0 | 0 | 0 | 0 | 1 | 0 |  |
|  | **d** | **0** | 0 | 0 | 0 | 0 | 0 | 1 | 1 | 0 | 0 | 0 | 0 | 0 | 0 |  |
|  | **e** | **0** | 0 | 0 | 0 | 0 | 0 | 1 | 0 | 0 | 0 | 0 | 0 | 0 | 0 |  |
|  | **f** | **1** | 1 | 2 | 1 | 1 | 1 | 1 | 1 | 1 | 1 | 0 | 1 | 1 | 1 |  |
|  | **g** | **1** | 1 | 2 | 1 | 1 | 1 | 1 | 1 | 1 | 1 | 1 | 1 | 1 | 1 |  |
|  | **h** | **0** | 0 | 0 | 0 | 0 | 0 | 1 | 1 | 0 | 0 | 0 | 0 | 0 | 0 |  |
|  | **i** | **1** | 1 | 0 | 1 | 1 | 1 | 1 | 1 | 0 | 1 | 0 | 1 | 1 | 1 |  |
|  | **j** | **1** | 1 | 0 | 1 | 1 | 1 | 1 | 1 | 1 | 1 | 2 | 1 | 1 | 1 |  |
|  | **k** | **0** | 0 | 0 | 0 | 0 | 0 | 1 | 1 | 0 | 0 | 0 | 0 | 0 | 0 |  |
|  | **l** | **1** | 1 | 1 | 1 | 1 | 1 | 1 | 1 | 1 | 1 | 1 | 1 | 1 | 0 |  |
|  | **m** | **1** | 1 | 0 | 1 | 1 | 1 | 1 | 1 | 1 | 1 | 1 | 1 | 1 | 1 |  |
|  | **n** | **1** | 1 | 0 | 1 | 1 | 1 | 1 | 1 | 0 | 1 | 0 | 1 | 1 | 0 |  |
|  | **o** | **1** | 1 | 1 | 1 | 1 | 1 | 1 | 1 | 1 | 1 | 0 | 1 | 1 | 1 |  |
|  | **p** | **1** | 1 | 1 | 1 | 1 | 1 | 1 | 1 | 0 | 1 | 0 | 1 | 0 | 1 |  |
|  | **q** | **0** | 0 | 0 | 0 | 0 | 0 | 1 | 1 | 0 | 0 | 0 | 0 | 1 | 0 |  |
|  | **r** | **0** | 0 | 0 | 0 | 0 | 0 | 1 | 0 | 0 | 0 | 0 | 0 | 0 | 0 |  |
|  | **s** | **1** | 1 | 0 | 1 | 1 | 1 | 1 | 1 | 0 | 1 | 0 | 1 | 1 | 0 |  |
|  | **t** | **1** | 1 | 0 | 1 | 1 | 1 | 1 | 1 | 0 | 1 | 1 | 1 | 1 | 1 |  |
|  | **u** | **1** | 1 | 1 | 1 | 1 | 1 | 1 | 1 | 1 | 1 | 2 | 1 | 1 | 1 |  |
|  | **v** | **0** | 0 | 0 | 0 | 2 | 0 | 1 | 2 | 0 | 0 | 0 | 0 | 2 | 0 |  |
|  | **w** | **1** | 0 | 0 | 0 | 2 | 2 | 1 | 1 | 0 | 1 | 2 | 1 | 0 | 1 |  |
|  | **x** | **1** | 1 | 1 | 1 | 1 | 1 | 1 | 1 | 1 | 1 | 1 | 1 | 1 | 1 |  |
|  |  |  | **P1** | **P2** | **P3** | **P4** | **P5**^c^ | **P6** | **P7** | **P8** | **P9** | **P11** | **P12** | **P13** | **P14** | **P15** |
| **Method a** | **a** | **1** | 1 | 1 |  |  | 1 |  |  | 1 | 1 | 1 |  |  |  |  |
|  | **b** | **0** | 0 | 0 |  |  | 1 |  |  | 0 | 0 | 0 |  |  |  |  |
|  | **c** | **0** | 0 | 0 |  |  | 1 |  |  | 0 | 0 | 0 |  |  |  |  |
|  | **d** | **0** | 0 | 0 |  |  | 0 |  |  | 1 | 0 | 0 |  |  |  |  |
|  | **e** | **0** | 0 | 0 |  |  | 1 |  |  | 0 | 0 | 0 |  |  |  |  |
|  | **f** | **1** | 1 | 1 |  |  | 1 |  |  | 1 | 1 | 1 |  |  |  |  |
|  | **g** | **1** | 1 | 1 |  |  | 1 |  |  | 1 | 1 | 1 |  |  |  |  |
|  | **h** | **0** | 0 | 0 |  |  | 1 |  |  | 0 | 0 | 0 |  |  |  |  |
|  | **i** | **1** | 1 | 1 |  |  | 1 |  |  | 1 | 1 | 1 |  |  |  |  |
|  | **j** | **1** | 1 | 1 |  |  | 1 |  |  | 1 | 1 | 1 |  |  |  |  |
|  | **k** | **0** | 0 | 0 |  |  | 0 |  |  | 0 | 0 | 0 |  |  |  |  |
|  | **l** | **1** | 1 | 1 |  |  | 1 |  |  | 1 | 1 | 1 |  |  |  |  |
|  | **m** | **1** | 1 | 1 |  |  | 1 |  |  | 1 | 1 | 1 |  |  |  |  |
|  | **n** | **1** | 1 | 1 |  |  | 1 |  |  | 1 | 1 | 1 |  |  |  |  |
|  | **o** | **1** | 1 | 1 |  |  | 1 |  |  | 1 | 1 | 1 |  |  |  |  |
|  | **p** | **1** | 1 | 0 |  |  | 1 |  |  | 0 | 0 | 0 |  |  |  |  |
|  | **q** | **0** | 0 | 0 |  |  | 0 |  |  | 0 | 0 | 0 |  |  |  |  |
|  | **r** | **0** | 0 | 0 |  |  | 1 |  |  | 0 | 0 | 0 |  |  |  |  |
|  | **s** | **1** | 1 | 1 |  |  | 1 |  |  | 0 | 0 | 2 |  |  |  |  |
|  | **t** | **1** | 1 | 1 |  |  | 1 |  |  | 1 | 1 | 1 |  |  |  |  |
|  | **u** | **1** | 1 | 0 |  |  | 1 |  |  | 0 | 0 | 0 |  |  |  |  |
|  | **v** | **0** | 0 | 2 |  |  | 0 |  |  | 1 | 0 | 0 |  |  |  |  |
|  | **w** | **1** | 1 | 2 |  |  | 1 |  |  | 1 | 0 | 0 |  |  |  |  |
|  | **x** | **1** | 1 | 1 |  |  | 1 |  |  | 1 | 1 | 1 |  |  |  |  |
|  |  |  | **P1** | **P2** | **P3** | **P4** | **P5** | **P6** | **P7** | **P8** | **P9** | **P11** | **P12** | **P13** | **P14** | **P15** |
| **Method 3** | **a** | **1** | 1 | 1 |  |  |  |  | 1 |  | 1 |  | 1 |  | 1 | 1 |
|  | **b** | **0** | 0 | 0 |  |  |  |  | 2 |  | 0 |  | 0 |  | 0 | 1 |
|  | **c** | **0** | 0 | 0 |  |  |  |  | 0 |  | 1 |  | 0 |  | 2 | 1 |
|  | **d** | **0** | 0 | 0 |  |  |  |  | 0 |  | 1 |  | 0 |  | 0 | 0 |
|  | **e** | **0** | 0 | 0 |  |  |  |  | 1 |  | 2 |  | 1 |  | 1 | 1 |
|  | **f** | **1** | 0 | 1 |  |  |  |  | 1 |  | 1 |  | 1 |  | 1 | 1 |
|  | **g** | **1** | 1 | 1 |  |  |  |  | 1 |  | 1 |  | 1 |  | 1 | 1 |
|  | **h** | **0** | 0 | 0 |  |  |  |  | 0 |  | 0 |  | 0 |  | 0 | 0 |
|  | **i** | **1** | 0 | 1 |  |  |  |  | 1 |  | 1 |  | 1 |  | 0 | 1 |
|  | **j** | **1** | 1 | 1 |  |  |  |  | 0 |  | 1 |  | 1 |  | 1 | 1 |
|  | **k** | **0** | 0 | 0 |  |  |  |  | 0 |  | 0 |  | 1 |  | 2 | 0 |
|  | **l** | **1** | 1 | 1 |  |  |  |  | 1 |  | 1 |  | 1 |  | 1 | 1 |
|  | **m** | **1** | 1 | 1 |  |  |  |  | 1 |  | 1 |  | 1 |  | 0 | 1 |
|  | **n** | **1** | 1 | 1 |  |  |  |  | 1 |  | 1 |  | 1 |  | 1 | 1 |
|  | **o** | **1** | 1 | 1 |  |  |  |  | 1 |  | 1 |  | 1 |  | 1 | 1 |
|  | **p** | **1** | 0 | 1 |  |  |  |  | 1 |  | 0 |  | 1 |  | 0 | 1 |
|  | **q** | **0** | 0 | 0 |  |  |  |  | 0 |  | 1 |  | 0 |  | 0 | 0 |
|  | **r** | **0** | 0 | 1 |  |  |  |  | 1 |  | 1 |  | 1 |  | 1 | 1 |
|  | **s** | **1** | 0 | 1 |  |  |  |  | 1 |  | 1 |  | 1 |  | 1 | 1 |
|  | **t** | **1** | 1 | 1 |  |  |  |  | 1 |  | 1 |  | 1 |  | 1 | 1 |
|  | **u** | **1** | 1 | 1 |  |  |  |  | 1 |  | 1 |  | 1 |  | 1 | 1 |
|  | **v** | **0** | 0 | 1 |  |  |  |  | 1 |  | 0 |  | 1 |  | 1 | 0 |
|  | **w** | **1** | 0 | 0 |  |  |  |  | 0 |  | 0 |  | 1 |  | 0 | 1 |
|  | **x** | **1** | 1 | 1 |  |  |  |  | 1 |  | 1 |  | 1 |  | 1 | 1 |
|  |  |  | **P1** | **P2** | **P3** | **P4** | **P5** | **P6** | **P7** | **P8** | **P9** | **P11** | **P12** | **P13** | **P14** | **P15** |
| **Method 4** | **a** | **1** | 1 | 1 | 1 |  | 1 |  | 0 |  | 1 |  | 1 | 1 | 1 | 1 |
|  | **b** | **0** | 0 | 0 | 0 |  | 0 |  | 0 |  | 0 |  | 0 | 0 | 0 | 0 |
|  | **c** | **0** | 0 | 0 | 0 |  | 0 |  | 0 |  | 0 |  | 0 | 0 | 0 | 0 |
|  | **d** | **0** | 0 | 0 | 2 |  | 0 |  | 0 |  | 2 |  | 0 | 1 | 0 | 0 |
|  | **e** | **0** | 0 | 0 | 0 |  | 0 |  | 0 |  | 0 |  | 0 | 1 | 0 | 0 |
|  | **f** | **1** | 1 | 1 | 1 |  | 1 |  | 1 |  | 1 |  | 1 | 1 | 1 | 1 |
|  | **g** | **1** | 1 | 1 | 1 |  | 1 |  | 1 |  | 1 |  | 1 | 1 | 1 | 1 |
|  | **h** | **0** | 0 | 0 | 0 |  | 0 |  | 0 |  | 0 |  | 0 | 0 | 0 | 0 |
|  | **i** | **1** | 1 | 1 | 1 |  | 1 |  | 1 |  | 1 |  | 1 | 1 | 1 | 1 |
|  | **j** | **1** | 1 | 1 | 1 |  | 1 |  | 1 |  | 1 |  | 1 | 1 | 1 | 1 |
|  | **k** | **0** | 0 | 0 | 0 |  | 0 |  | 0 |  | 0 |  | 0 | 2 | 0 | 0 |
|  | **l** | **1** | 1 | 1 | 1 |  | 1 |  | 1 |  | 1 |  | 1 | 1 | 1 | 1 |
|  | **m** | **1** | 1 | 1 | 1 |  | 1 |  | 1 |  | 0 |  | 1 | 1 | 1 | 1 |
|  | **n** | **1** | 1 | 1 | 1 |  | 1 |  | 1 |  | 1 |  | 1 | 1 | 1 | 1 |
|  | **o** | **1** | 1 | 1 | 1 |  | 1 |  | 1 |  | 1 |  | 1 | 1 | 1 | 1 |
|  | **p** | **1** | 1 | 2 | 1 |  | 2 |  | 1 |  | 1 |  | 1 | 1 | 1 | 1 |
|  | **q** | **0** | 0 | 0 | 0 |  | 0 |  | 0 |  | 0 |  | 0 | 1 | 0 | 0 |
|  | **r** | **0** | 0 | 0 | 0 |  | 0 |  | 0 |  | 0 |  | 0 | 0 | 0 | 0 |
|  | **s** | **1** | 1 | 1 | 1 |  | 1 |  | 1 |  | 1 |  | 1 | 1 | 1 | 1 |
|  | **t** | **1** | 1 | 1 | 1 |  | 1 |  | 1 |  | 1 |  | 1 | 1 | 1 | 1 |
|  | **u** | **1** | 1 | 1 | 1 |  | 1 |  | 1 |  | 1 |  | 1 | 1 | 1 | 1 |
|  | **v** | **0** | 0 | 1 | 0 |  | 0 |  | 0 |  | 2 |  | 0 | 0 | 2 | 1 |
|  | **w** | **1** | 1 | 2 | 1 |  | 1 |  | 1 |  | 0 |  | 1 | 0 | 0 | 1 |
|  | **x** | **1** | 1 | 1 | 1 |  | 1 |  | 1 |  | 1 |  | 1 | 1 | 1 | 1 |
|  |  |  | **P1** | **P2** | **P3** | **P4** | **P5** | **P6** | **P7** | **P8** | **P9**^c^ | **P11** | **P12** | **P13** | **P14** | **P15** |
| **Method 5** | **a** | **1** | 1 | 1 |  |  | 1 |  | 1 |  | 1 |  | 1 | 1 | 1 |  |
|  | **b** | **0** | 0 | 0 |  |  | 0 |  | 0 |  | 0 |  | 1 | 0 | 0 |  |
|  | **c** | **0** | 0 | 2 |  |  | 0 |  | 1 |  | 1 |  | 0 | 0 | 0 |  |
|  | **d** | **0** | 0 | 0 |  |  | 2 |  | 0 |  | 2 |  | 0 | 0 | 0 |  |
|  | **e** | **0** | 0 | 0 |  |  | 0 |  | 0 |  | 2 |  | 0 | 0 | 0 |  |
|  | **f** | **1** | 1 | 1 |  |  | 1 |  | 1 |  | 1 |  | 1 | 1 | 1 |  |
|  | **g** | **1** | 1 | 1 |  |  | 1 |  | 1 |  | 1 |  | 1 | 1 | 1 |  |
|  | **h** | **0** | 0 | 0 |  |  | 0 |  | 1 |  | 2 |  | 0 | 0 | 0 |  |
|  | **i** | **1** | 1 | 1 |  |  | 1 |  | 1 |  | 1 |  | 1 | 1 | 1 |  |
|  | **j** | **1** | 1 | 1 |  |  | 1 |  | 0 |  | 1 |  | 1 | 1 | 1 |  |
|  | **k** | **0** | 0 | 0 |  |  | 0 |  | 0 |  | 2 |  | 0 | 0 | 0 |  |
|  | **l** | **1** | 1 | 1 |  |  | 1 |  | 1 |  | 1 |  | 1 | 1 | 1 |  |
|  | **m** | **1** | 1 | 1 |  |  | 1 |  | 1 |  | 1 |  | 1 | 1 | 1 |  |
|  | **n** | **1** | 1 | 1 |  |  | 1 |  | 1 |  | 1 |  | 1 | 1 | 1 |  |
|  | **o** | **1** | 1 | 1 |  |  | 1 |  | 1 |  | 1 |  | 1 | 1 | 1 |  |
|  | **p** | **1** | 1 | 1 |  |  | 1 |  | 2 |  | 1 |  | 1 | 1 | 1 |  |
|  | **q** | **0** | 0 | 2 |  |  | 0 |  | 0 |  | 0 |  | 0 | 1 | 0 |  |
|  | **r** | **0** | 0 | 0 |  |  | 0 |  | 1 |  | 2 |  | 0 | 0 | 0 |  |
|  | **s** | **1** | 1 | 1 |  |  | 1 |  | 1 |  | 1 |  | 1 | 0 | 1 |  |
|  | **t** | **1** | 1 | 1 |  |  | 1 |  | 1 |  | 1 |  | 1 | 1 | 1 |  |
|  | **u** | **1** | 0 | 1 |  |  | 0 |  | 1 |  | 1 |  | 2 | 0 | 1 |  |
|  | **v** | **0** | 0 | 0 |  |  | 1 |  | 0 |  | 2 |  | 0 | 1 | 0 |  |
|  | **w** | **1** | 1 | 1 |  |  | 1 |  | 0 |  | 1 |  | 1 | 0 | 1 |  |
|  | **x** | **1** | 1 | 1 |  |  | 1 |  | 1 |  | 1 |  | 1 | 1 | 1 |  |
|  |  |  | **P1** | **P2** | **P3** | **P4** | **P5** | **P6** | **P7** | **P8** | **P9** | **P11** | **P12** | **P13** | **P14** | **P15** |
| **Method 6** | **a** | **1** | 1 | 1 |  |  | 1 |  | 1 |  | 1 |  | 1 | 1 | 1 | 1 |
|  | **b** | **0** | 0 | 0 |  |  | 0 |  | 0 |  | 0 |  | 0 | 0 | 0 | 2 |
|  | **c** | **0** | 0 | 0 |  |  | 0 |  | 1 |  | 0 |  | 0 | 0 | 0 | 2 |
|  | **d** | **0** | 0 | 0 |  |  | 0 |  | 0 |  | 0 |  | 2 | 0 | 2 | 0 |
|  | **e** | **0** | 0 | 0 |  |  | 0 |  | 0 |  | 0 |  | 0 | 0 | 0 | 0 |
|  | **f** | **1** | 1 | 1 |  |  | 1 |  | 1 |  | 1 |  | 1 | 1 | 1 | 1 |
|  | **g** | **1** | 1 | 1 |  |  | 1 |  | 1 |  | 1 |  | 1 | 1 | 1 | 1 |
|  | **h** | **0** | 0 | 0 |  |  | 0 |  | 1 |  | 0 |  | 0 | 0 | 0 | 0 |
|  | **i** | **1** | 1 | 1 |  |  | 1 |  | 1 |  | 1 |  | 1 | 1 | 1 | 1 |
|  | **j** | **1** | 1 | 1 |  |  | 1 |  | 1 |  | 1 |  | 1 | 1 | 1 | 1 |
|  | **k** | **0** | 0 | 0 |  |  | 0 |  | 1 |  | 0 |  | 0 | 0 | 2 | 0 |
|  | **l** | **1** | 1 | 1 |  |  | 1 |  | 1 |  | 1 |  | 1 | 1 | 1 | 1 |
|  | **m** | **1** | 1 | 1 |  |  | 1 |  | 1 |  | 1 |  | 1 | 1 | 1 | 1 |
|  | **n** | **1** | 1 | 1 |  |  | 1 |  | 1 |  | 1 |  | 1 | 1 | 1 | 1 |
|  | **o** | **1** | 1 | 1 |  |  | 1 |  | 1 |  | 1 |  | 1 | 1 | 1 | 1 |
|  | **p** | **1** | 2 | 2 |  |  | 1 |  | 0 |  | 1 |  | 1 | 1 | 1 | 1 |
|  | **q** | **0** | 0 | 0 |  |  | 0 |  | 0 |  | 0 |  | 0 | 0 | 2 | 0 |
|  | **r** | **0** | 0 | 0 |  |  | 0 |  | 1 |  | 0 |  | 0 | 0 | 0 | 0 |
|  | **s** | **1** | 1 | 1 |  |  | 1 |  | 1 |  | 1 |  | 1 | 1 | 1 | 1 |
|  | **t** | **1** | 1 | 1 |  |  | 1 |  | 1 |  | 1 |  | 1 | 1 | 1 | 1 |
|  | **u** | **1** | 1 | 1 |  |  | 1 |  | 1 |  | 1 |  | 1 | 0 | 1 | 1 |
|  | **v** | **0** | 0 | 2 |  |  | 0 |  | 0 |  | 0 |  | 0 | 2 | 2 | 0 |
|  | **w** | **1** | 2 | 2 |  |  | 1 |  | 0 |  | 0 |  | 1 | 0 | 1 | 2 |
|  | **x** | **1** | 1 | 1 |  |  | 1 |  | 1 |  | 1 |  | 1 | 1 | 1 | 1 |

^a^1 for positive, 0 for negative and 2 for indeterminate or missing value

^b^The shaded cells indicate indeterminate results or results that are discordant with regard to the expected results

^c^Results excluded from the data analysis
