# Supplementary material for: Data processing of qualitative results from an interlaboratory comparison for the detection of “Flavescence dorée” phytoplasma: How the use of statistics can improve the reliability of the method validation process in plant pathology
Source: PLoS One. 2017 Apr 6;12(4):e0175247. doi: 10.1371/journal.pone.0175247 (PMC5383269; doi:10.1371/journal.pone.0175247)
Supplement: S6 Table — (DOC) [file pone.0175247.s006.doc]

*TABLE S6. Results submitted by the different laboratories during the collaborative study for the second stage of the evaluation.*

| **Method** | **Sample code** | **Number of positive results obtained by the different laboratories^a^** | | | | |
| --- | --- | --- | --- | --- | --- | --- |
|  |  | **P1** | **P2** | **P7** | **P12** | **P14** |
| **Method 1** | **A1** | 5 | 1 | 5 | 5 | 5 |
|  | **A2** | 5 | 3 | 5 | 5 | 5 |
|  | **A3** | 5 | 4 | 5 | 5 | 5 |
|  | **A4** | 4 | 2 | 5 | 3 | 2 |
|  | **A5** | 3 | 2 | 5 | 4 | 4 |
|  | **B1** | 5 | 3 | 5 | 5 | 5 |
|  | **B2** | 5 | 4 | 5 | 5 | 5 |
|  | **B3** | 2 | 3 | 4 | 2 | 1 |
|  | **B4** | 1 | 0 | 1 | 0 | 0 |
|  | **B5** | 1 | 1 | 3 | 2 | 1 |
|  | **C1** | 5 | 5 | 3 | 5 | 5 |
|  | **C2** | 5 | 3 | 3 | 5 | 5 |
|  | **C3** | 5 | 2 | 3 | 5 | 5 |
|  | **C4** | 5 | 2 | 3 | 5 | 5 |
|  | **C5** | 5 | 2 | 5 | 5 | 5 |
|  |  | **P1** | **P2** | **P3** | **P4** | **P5** |
| **Method 2** | **A1** | 5 | 0 | 0 | 5 | 0 |
|  | **A2** | 5 | 0 | 0 | 5 | 0 |
|  | **A3** | 2 | 0 | 0 | 5 | 0 |
|  | **A4** | 1 | 0 | 0 | 3 | 0 |
|  | **A5** | 0 | 0 | 0 | 0 | 0 |
|  | **B1** | 5 | 0 | 0 | 5 | 4 |
|  | **B2** | 5 | 0 | 0 | 4 | 0 |
|  | **B3** | 5 | 0 | 0 | 3 | 0 |
|  | **B4** | 4 | 0 | 0 | 2 | 0 |
|  | **B5** | 3 | 0 | 0 | 0 | 0 |
|  | **C1** | 0 | 1 | 0 | 5 | 2 |
|  | **C2** | 5 | 0 | 0 | 5 | 0 |
|  | **C3** | 5 | 0 | 0 | 5 | 0 |
|  | **C4** | 5 | 0 | 0 | 5 | 0 |
|  | **C5** | 4 | 0 | 1 | 5 | 2 |
|  |  | **P1** | **P2** | **P3** | **P4** | **P5**^c^ |
| **Method a^b^** | **A1** | 5 | 1 |  |  |  |
|  | **A2** | 5 | 4 |  |  |  |
|  | **A3** | 5 | 4 |  |  |  |
|  | **A4** | 4 | 4 |  |  |  |
|  | **A5** | 3 | 5 |  |  |  |
|  | **B1** | 5 | 4 |  |  |  |
|  | **B2** | 4 | 4 |  |  |  |
|  | **B3** | 2 | 5 |  |  |  |
|  | **B4** | 1 | 4 |  |  |  |
|  | **B5** | 1 | 5 |  |  |  |
|  | **C1** | 5 | 3 |  |  |  |
|  | **C2** | 5 | 4 |  |  |  |
|  | **C3** | 5 | 3 |  |  |  |
|  | **C4** | 5 | 3 |  |  |  |
|  | **C5** | 5 | 3 |  |  |  |
|  |  | **P1** | **P2** | **P3** | **P4** | **P5** |
| **Method 3** | **A1** | 5 | 1 | 5 | 5 | 5 |
|  | **A2** | 5 | 3 | 5 | 5 | 5 |
|  | **A3** | 5 | 4 | 5 | 5 | 5 |
|  | **A4** | 5 | 1 | 5 | 5 | 5 |
|  | **A5** | 5 | 2 | 5 | 5 | 5 |
|  | **B1** | 5 | 5 | 4 | 5 | 5 |
|  | **B2** | 5 | 3 | 4 | 5 | 5 |
|  | **B3** | 5 | 2 | 5 | 5 | 5 |
|  | **B4** | 4 | 5 | 4 | 5 | 5 |
|  | **B5** | 2 | 2 | 3 | 1 | 3 |
|  | **C1** | 0 | 3 | 5 | 5 | 0 |
|  | **C2** | 5 | 1 | 5 | 5 | 5 |
|  | **C3** | 5 | 4 | 5 | 5 | 5 |
|  | **C4** | 5 | 3 | 3 | 5 | 5 |
|  | **C5** | 5 | 2 | 0 | 5 | 5 |
|  |  | **P1** | **P2** | **P3** | **P4** | **P5** |
| **Method 4** | **A1** | 5 | 2 | 5 | 5 | 5 |
|  | **A2** | 5 | 4 | 5 | 5 | 5 |
|  | **A3** | 5 | 5 | 5 | 5 | 5 |
|  | **A4** | 5 | 3 | 5 | 5 | 5 |
|  | **A5** | 5 | 5 | 5 | 5 | 5 |
|  | **B1** | 5 | 2 | 3 | 5 | 5 |
|  | **B2** | 5 | 5 | 5 | 5 | 5 |
|  | **B3** | 4 | 5 | 4 | 5 | 5 |
|  | **B4** | 3 | 1 | 4 | 2 | 4 |
|  | **B5** | 2 | 0 | 0 | 2 | 3 |
|  | **C1** | 5 | 5 | 0 | 5 | 5 |
|  | **C2** | 5 | 5 | 5 | 5 | 5 |
|  | **C3** | 5 | 5 | 5 | 5 | 4 |
|  | **C4** | 5 | 5 | 5 | 5 | 5 |
|  | **C5** | 5 | 5 | 3 | 5 | 5 |
|  |  | **P1** | **P2** | **P3** | **P4** | **P5** |
| **Method 5** | **A1** | 5 | 5 | 5 | 5 | 5 |
|  | **A2** | 5 | 5 | 5 | 5 | 5 |
|  | **A3** | 5 | 5 | 5 | 5 | 5 |
|  | **A4** | 5 | 5 | 5 | 5 | 5 |
|  | **A5** | 5 | 5 | 5 | 5 | 5 |
|  | **B1** | 5 | 5 | 5 | 5 | 5 |
|  | **B2** | 5 | 5 | 5 | 5 | 5 |
|  | **B3** | 5 | 3 | 5 | 5 | 5 |
|  | **B4** | 5 | 3 | 4 | 4 | 3 |
|  | **B5** | 5 | 2 | 2 | 2 | 4 |
|  | **C1** | 5 | 5 | 5 | 5 | 5 |
|  | **C2** | 5 | 5 | 5 | 5 | 5 |
|  | **C3** | 5 | 5 | 5 | 5 | 5 |
|  | **C4** | 5 | 5 | 5 | 5 | 5 |
|  | **C5** | 5 | 5 | 5 | 5 | 5 |
|  |  | **P1** | **P2** | **P3** | **P4** | **P5** |
| **Method 6** | **A1** | 5 | 5 | 5 | 5 | 5 |
|  | **A2** | 5 | 5 | 5 | 5 | 5 |
|  | **A3** | 5 | 5 | 5 | 5 | 5 |
|  | **A4** | 4 | 5 | 5 | 5 | 5 |
|  | **A5** | 3 | 5 | 4 | 5 | 4 |
|  | **B1** | 5 | 5 | 5 | 5 | 5 |
|  | **B2** | 5 | 3 | 5 | 5 | 5 |
|  | **B3** | 4 | 4 | 2 | 5 | 3 |
|  | **B4** | 3 | 3 | 3 | 3 | 2 |
|  | **B5** | 1 | 2 | 1 | 2 | 2 |
|  | **C1** | 5 | 5 | 5 | 5 | 5 |
|  | **C2** | 5 | 4 | 5 | 5 | 5 |
|  | **C3** | 5 | 5 | 5 | 5 | 5 |
|  | **C4** | 5 | 5 | 5 | 5 | 5 |
|  | **C5** | 4 | 5 | 5 | 5 | 5 |

^a^Each sample was analyzed five times by each laboratory

^b^Method Ma was implemented only by laboratories P1 and P2
